# Local anisotropy in mineralized fibrocartilage and subchondral bone beneath the tendon-bone interface

**DOI:** 10.1038/s41598-021-95917-4

**Published:** 2021-08-16

**Authors:** Alexandra Tits, Erwan Plougonven, Stéphane Blouin, Markus A. Hartmann, Jean-François Kaux, Pierre Drion, Justin Fernandez, G. Harry van Lenthe, Davide Ruffoni

**Affiliations:** 1 Mechanics of Biological and Bioinspired Materials Laboratory, Department of Aerospace and Mechanical Engineering, University of Liège, Quartier Polytech 1, Allée de la Découverte 9, 4000 Liège, Belgium; 2 Chemical Engineering Department, University of Liège, Liège, Belgium; 3 Ludwig Boltzmann Institute of Osteology, Hanusch Hospital of OEGK and AUVA Trauma Centre Meidling, 1st Medical Department Hanusch Hospital, Vienna, Austria; 4 Department of Physical Medicine and Sports Traumatology, University of Liège and University Hospital of Liège, Liège, Belgium; 5 Experimental Surgery Unit, GIGA and Credec, University of Liege, Liege, Belgium; 6 Auckland Bioengineering Institute and Department of Engineering Science, University of Auckland, Auckland, New Zealand; 7 Department of Mechanical Engineering, KU Leuven, Leuven, Belgium

**Keywords:** Biomedical engineering, X-ray tomography, Bone, Tissues

## Abstract

The enthesis allows the insertion of tendon into bone thanks to several remarkable strategies. This complex and clinically relevant location often features a thin layer of fibrocartilage sandwiched between tendon and bone to cope with a highly heterogeneous mechanical environment. The main purpose of this study was to investigate whether mineralized fibrocartilage and bone close to the enthesis show distinctive three-dimensional microstructural features, possibly to enable load transfer from tendon to bone. As a model, the Achilles tendon-calcaneus bone system of adult rats was investigated with histology, backscattered electron imaging and micro-computed tomography. The microstructural porosity of bone and mineralized fibrocartilage in different locations including enthesis fibrocartilage, periosteal fibrocartilage and bone away from the enthesis was characterized. We showed that calcaneus bone presents a dedicated protrusion of low porosity where the tendon inserts. A spatially resolved analysis of the trabecular network suggests that such protrusion may promote force flow from the tendon to the plantar ligament, while partially relieving the trabecular bone from such a task. Focusing on the tuberosity, highly specific microstructural aspects were highlighted. Firstly, the interface between mineralized and unmineralized fibrocartilage showed the highest roughness at the tuberosity, possibly to increase failure resistance of a region carrying large stresses. Secondly, fibrochondrocyte lacunae inside mineralized fibrocartilage, in analogy with osteocyte lacunae in bone, had a predominant alignment at the enthesis and a rather random organization away from it. Finally, the network of subchondral channels inside the tuberosity was highly anisotropic when compared to contiguous regions. This dual anisotropy of subchondral channels and cell lacunae at the insertion may reflect the alignment of the underlying collagen network. Our findings suggest that the microstructure of fibrocartilage may be linked with the loading environment. Future studies should characterize those microstructural aspects in aged and or diseased conditions to elucidate the poorly understood role of bone and fibrocartilage in enthesis-related pathologies.

## Introduction

A crucial requirement of the musculoskeletal apparatus is the transmission of forces from tendons to bone. This can be a challenging task owing to the strong differences in composition, structure and material behavior between the soft tissue and the hard mineralized bone, making connecting regions vulnerable to high stresses, which may trigger failure upon repeated loading^1^. Consequently, the integration of tendons into bone often occurs through a specialized multi-material region called enthesis, which comprises fibrocartilage as an intermediate tissue to mitigate the incompatibilities between the two materials^2^. Entheses are traditionally subdivided into four contiguous regions: the tendon, the unmineralized fibrocartilage, the mineralized fibrocartilage and the bone. The inclusion of tendon and bone in the definition of entheses highlights the absence of a well-defined interface among these tissues^3^. From a compositional point of view, in contrast to tendon and bone, which are fibrous tissues based on collagen type I, enthesis fibrocartilage features a higher content of collagen type II and proteoglycans^4,5^. The latter, having a high affinity for water, are believed to provide fibrocartilage with improved resistance to compression^2^. The transition from tendon to unmineralized fibrocartilage is characterized by a large decrease in the diameter of the tendon fibers, which branches into smaller interface fibers in fibrocartilage^5,6^. Fibrocartilage fibers are somewhat less aligned along the tendon direction^7^ and splay out^5^, resulting in a broader attachment area. Before reaching bone, the fibrocartilage matrix gets reinforced by mineral crystals^8^. Rather than being flat, the interface between unmineralized and mineralized fibrocartilage is fairly wavy^9,10^ and the junction between mineralized fibrocartilage and bone is highly interlocked^2,11^. Such compositional and architectural modifications are considered remarkable adaptation strategies to improve the endurance and the robustness of entheses, enabling the transmission of loads even higher than body weight for millions of loading cycles^5,9,12–17^.

Despite a finely tuned and unique biomechanical behavior, entheses are of considerable clinical relevance for several reasons. They are vulnerable to overuse injuries^3,18^, rheumatic pathologies^19–21^ and degenerative changes^22,23^. Some of these conditions seem to be more prevalent in entheses subjected to intense mechanical loading^3^, and there is probably a relationship (yet poorly understood) between the highly heterogeneous local mechanical environment at the enthesis and the likelihood to develop enthesopathies^24^. Enthesis pathologies such as enthesitis are more complex to treat than pure tendon or bone lesions due to their histological and biomechanical characteristics and should be included into a potential general inflammatory context. From an orthopedic perspective, tendon traumatic or acute injuries, even if they do not directly involve the attachment region, often require the surgical reattachment of the soft tissue to the bone. However, the enthesis, unlike bone and to some extent tendon, has slow and limited regeneration ability^25–27^. As a consequence, surgical treatments can have a rather poor long-term outcome^28–31^*.* Furthermore, there is strong biomechanical and clinical evidence that failure can occur not only at the soft side of the enthesis but also within the subchondral bone beneath the insertion^32^, and even in the bone far away from the attachment region^33–35^. Those facts highlight the central role of bone in the anchoring process.

Bone has the unique ability to reorganize its structure and material properties according to the local mechanical environment^36,37^. Over the last years, many in vivo mechanobiological experiments have shown that tissue level mechanical forces are able to drive local bone formation and resorption^29,38–42^, leading to reconfigurations of cortical and trabecular bone morphology. In aged conditions or following implant insertion, the bone mechanosensory machine is disturbed, causing a decreased mechanoresponsiveness^43,44^, which may lead to bone loss. Although less understood, there are suggestions that even the mineralization process^37^, the orientation of the vascular canals^45,46^, the shape and arrangement of the osteocyte lacunae^47,48^ and, at smaller length scales, the organization of the osteocyte canalicular network^49,50^ may mirror the loading environment. The bone beneath the enthesis has the critical biomechanical task of receiving loads from the tendon and redistributing them away from the attachment point. Surprisingly, only little efforts have been made to characterize bone properties near insertions. Therefore, the main purpose of this study is to investigate bone microstructure close to the enthesis at multiple length scales, from whole bone down to cell lacunae. Such characterization is needed to improve our knowledge of bone adaptation close to soft tissue attachments, with perspectives on orthopedics and regenerative medicine.

**Figure 1:**
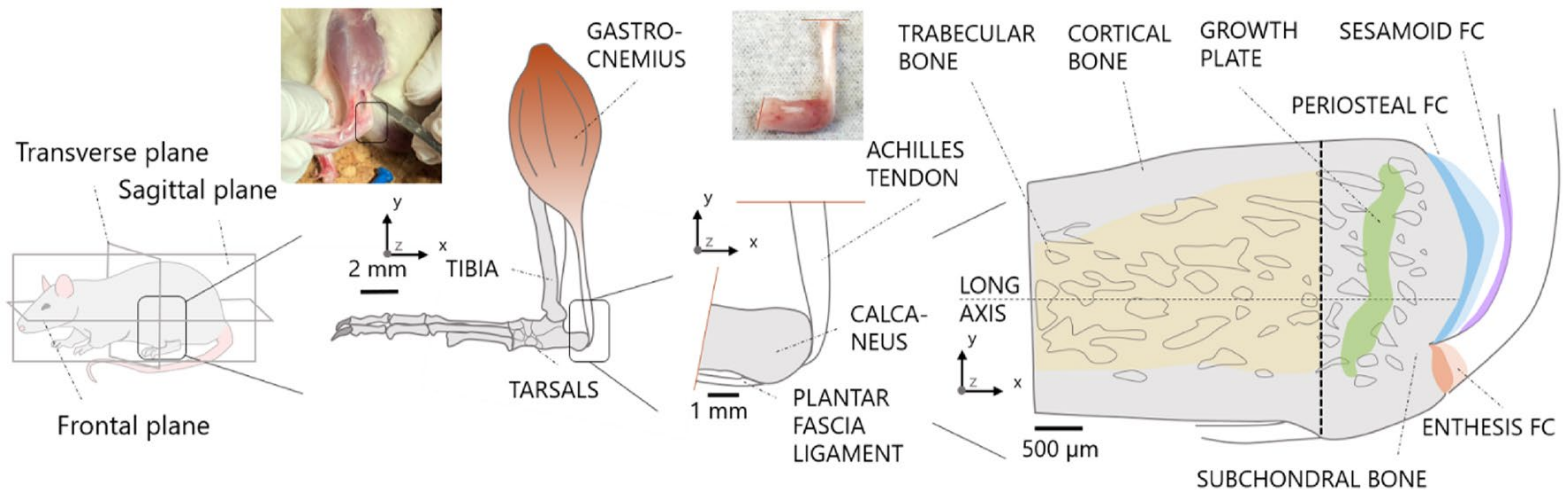
Schematic overview of the ankle joint. Several anatomical details and the different types of fibrocartilage required for joint functioning are highlighted. In addition to enthesis and periosteal fibrocartilage (FC), the tendon surface close to the enthesis is covered by a layer of sesamoid fibrocartilage, likely to protect the tendon by providing resistance against compressive and shear stresses coming from the contact with the bone. Unmineralized and mineralized fibrocartilages are shown in light and dark colors, respectively. Pictures of the tendon-bone construct were taken during sample extraction.

As a biological system, the Achilles tendon insertion into calcaneus bone is considered (Fig. 1). At this anatomical location, bone is bordered with two types of fibrocartilage^51,52^: the enthesis fibrocartilage, enabling the attachment of tendon into bone, and the periosteal fibrocartilage, which covers the bone surface above the insertion and facilitates the sliding between tendon and bone during joint movement (e.g. dorsiflexion). Here, we assume that bone shows distinctive features to cope with tendon loading, which are not present in regions away from the insertion. Specifically, we hypothesize that bone microstructure and microporosity beneath enthesis fibrocartilage differs from bone located below periosteal fibrocartilage, reflecting a distinct mechanical environment. Our core investigation is based on micro-computed tomography (micro-CT) at two different length scales, combined with advanced image processing. As entheses are complex regions comprising different tissues^18,96^, we used histology and backscattered electron imaging (BEI) to complement the micro-CT analysis with biological information on tissue type, location and organization. We performed a spatially resolved analysis of trabecular bone when approaching the enthesis. Then, we characterized the arrangement of subchondral channels and fibrochondrocyte lacunae within bone and fibrocartilage, respectively. Finally, we measured the local roughness of the interface anchoring unmineralized to mineralized fibrocartilage.

## Materials and methods

### Sample preparation, histology, backscattered electron imaging and micro-CT

Achilles tendon-calcaneus bone samples (n = 7) were carefully extracted from the posterior legs of 3-month-old male Sprague–Dawley rats (Fig. 1). Animal weight was 450 g ± 50 g and samples were available at the Liège University Hospital (CHU) in the framework of a sample organ donation program approved by the Animal Ethics Committee of the University of Liège (ULg IACUC-21-2340). The procedure was performed in accordance with relevant guidelines and regulations and reported in compliance with the ARRIVE guidelines. After extraction, two samples were prepared for histology. Tendon-bone specimens were fixed in 70% ethanol, dehydrated (baths of increasing ethanol concentration of 80–90–100%, 24 h each) and degreased by acetone. Subsequently, samples were embedded in poly-methylmethacrylate (PMMA). Sections with a thickness of approximately 3 µm were cut from the blocks with a hard tissue microtome (Leica SM2500, Nussloch, Germany) and stained either with Giemsa or with Goldner trichrome to distinguish fibrocartilage from bone. The sections were then visualized with a light microscope, also using polarized light (Axiophot, Zeiss, Oberkochen, Germany) equipped with a digital camera (Axiocam HRc, Zeiss). The rest block was used to perform BEI with 20 kV (Field Emission SEM Supra40, Zeiss) to distinguish mineralized from non-mineralized tissue. At the same time, the remaining five samples used for micro-CT imaging were dried overnight at room temperature directly after extraction and then glued on a custom three-dimensional printed support. Whole tendon-bone specimens were first scanned with micro-CT at a nominal isotropic voxel size of 5 μm. In brief, the machine (Skyscan 1272, Bruker, Belgium) was operated at tube voltage of 60 kV and current of 166 µA, in combination with a 0.25 mm thick aluminum filter. The samples were rotated over 180° with a rotation step of 0.2° (corresponding to 900 projections), with an exposure time of 2000 ms and a frame averaging of 4, leading to a scan time of approximately 3 h. Following the first scan at the whole bone level, samples were fixed (70% ethanol, 24 h) and dehydrated (baths of increasing ethanol concentration of 80–90–100%, 24 h each) before subsequent embedding in epoxy resin (EpoThin 2 Resin, Buehler, Germany). The size of the samples along the cranio-caudal direction (Fig. 1) was reduced by performing a transverse cut with a manual saw, to enable scanning at a higher magnification of a smaller bone region adjacent to the periosteal and enthesis fibrocartilages. Samples were embedded to facilitate cutting and to minimize motion artifacts which can hamper image quality when scanning at high resolution and long scanning time. Embedded bone samples were then imaged with the same micro-CT machine at a nominal isotropic resolution of 1.25 µm, using a 0.5 mm aluminum filter combined with voltage and current of 55 kV and 181 µA, respectively. Again 900 projections were acquired with an exposure of 6000 ms and frame averaging of 2, for a scan duration of about 10 h per sample. Images were reconstructed using a filtered back projection algorithm, including ring artifact reduction and beam hardening correction^53^, as implemented in the reconstruction software of the scanner (NRecon v.1.7.5.2, Skyscan).

### Image processing and quantitative morphometry

#### Image alignment and segmentation

**Figure 2:**
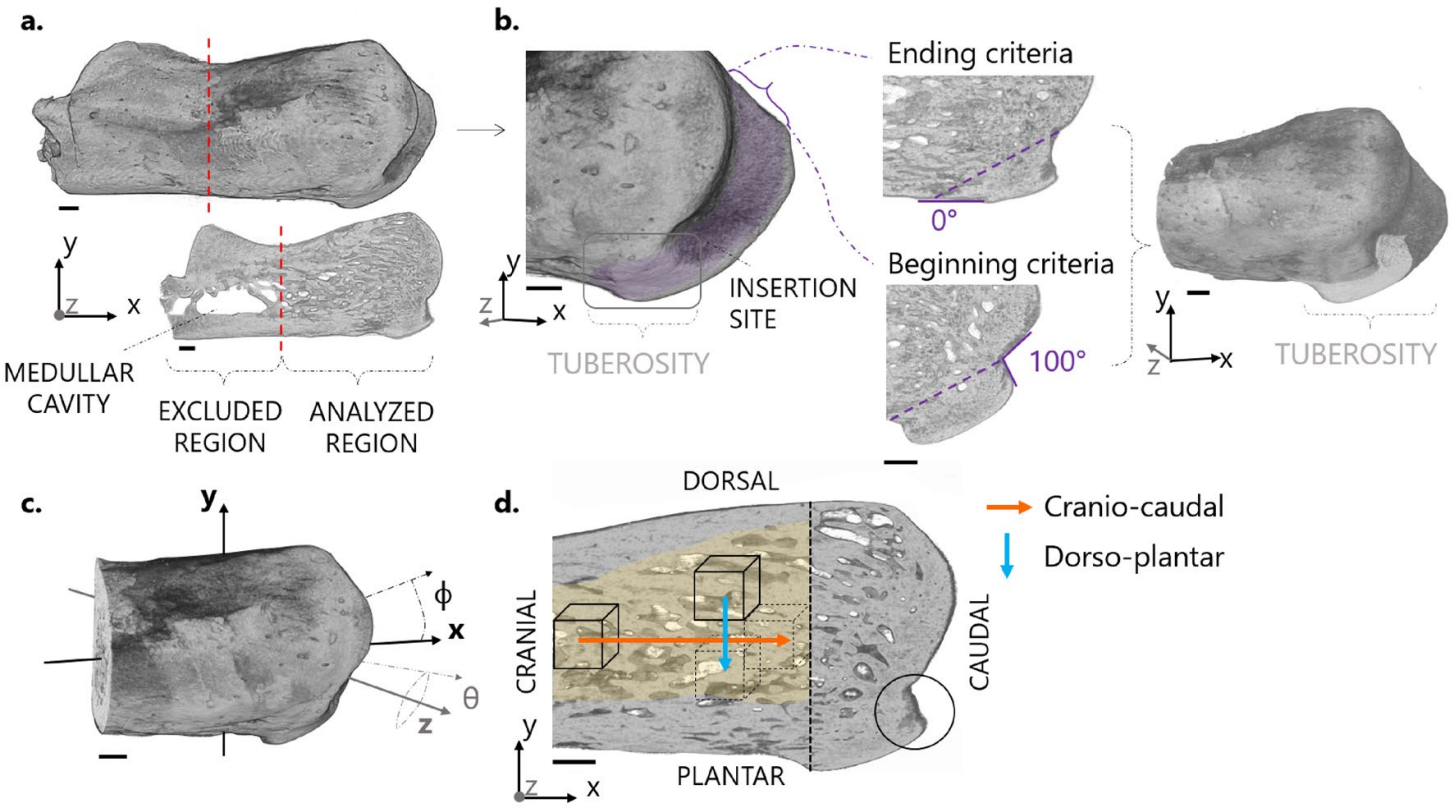
Image segmentation and reference coordinate system. (**a**) Three-dimensional rendering and typical sagittal section of a reconstructed and realigned calcaneus bone at 5 μm resolution. The cutting plane (red dashed lines) demarcates the analyzed region. (**b**) Cropped bone with a close up on the insertion site. The cross-sections considered for the tuberosity analysis are highlighted in purple. The anatomical landmarks to identify the tuberosity in the sagittal plane are also illustrated, together with the cutting orientation (dashed lines) used for the segmentation of the tuberosity. (**c**) Representation of the spherical coordinate system with the azimuthal (ϕ) and polar (θ) angles used to define the orientation of microstructural features. (**d**) Illustration of the cubic volumes of interest defined for the spatially resolved analysis of the trabecular network (the analyzed trabecular compartment is highlighted in yellow). Cubes were displaced along two directions: cranio-caudal (orange arrow) and dorso-plantar (blue arrow). Scale bars: 500 µm.

The reconstructed three-dimensional images of the whole tendon-bone complex were cropped along the transverse plane right before the medullar cavity (Fig. 2a). The virtual bones were then aligned along their three principal axes of inertia using BoneJ (v.1.4.3), a module of ImageJ (v.1.52a)^54,55^. Following this procedure, a distinct bony protrusion (referred as tuberosity) was revealed at the tendon attachment site, particularly evident when looking at sagittal sections (i.e., XY plane in Fig. 2b). The high-resolution scans were also aligned along the principal axes of inertia and slightly rotated in the sagittal plane so that the tuberosity of the low- and high-resolution images were co-aligned (Supplementary Fig. S1). To define the overall three-dimensional extent of the tuberosity, several consecutive cross-sections in the XY sagittal plane were inspected for anatomical landmarks in relation to the presence of the tendon, which was visible in the low-resolution scans (Supplementary Fig. S1): the tuberosity was considered to start as soon as it generated an angle of 100° with the superior periosteal region, and to end when there was no protrusion evident anymore on the plantar side of the bone (Fig. 2b). All subsequent image processing procedures were done using Matlab (R2018a; The Mathworks, USA), CTAn (v1.19.4.0, Skyscan) and Avizo (v.9.2.0, ThermoFisher Scientific). Before segmentation and quantitative analysis, images were smoothed with a three-dimensional Gaussian filter (square kernel 1.5 radius, 0.65 standard deviation). Filtering of raw micro-CT data is often required prior to segmentation to remove inherent signal noise. Gaussian filters are frequently used as they allow noise removal while keeping a satisfying contrast between bone and background^56^. Considering the good signal-to-noise ratio of our micro-CT measurements, only a limited amount of filtering was necessary. Fig. S2 recapitulates the impact of Gaussian blurring on noise reduction with representative cross sections of the micro-CT images at 1.25 µm and 5 µm voxel size. In both cases, all microstructural features of interest were well-preserved. After filtering, images were binarized using a global threshold calculated with Otsu’s method^57^, as implemented in CTAn. This is an iterative algorithm ideal for bimodal distributions of gray values which possess a valley between the two peaks^58^. It finds a threshold that maximizes the difference in mean values while minimizing the variance between voxels classified as bone and background. A graphical explanation of Otsu’s method is provided in Fig. S3.

#### Tuberosity and trabecular bone analysis

The bony tuberosity and the trabecular network were investigated using mainly the lower resolution (i.e. 5 µm) scans, analyzed with the software CTAn. The tuberosity was virtually separated from the remaining bone along an oblique plane, as illustrated in Fig. 2b. This region was quantified by measuring the overall tissue volume (i.e., tuberosity tissue volume, T.TV) and the orientation (T.θ, T.ϕ). Those angles describe the orientation of the tuberosity with respect to the principal axes of inertia of the bone (Fig. 2c). Additionally, the high-resolution scans were used to measure the tuberosity porosity (T.Po). The segmentation of the trabecular compartment from the remaining bone was performed using a customized multistep procedure: firstly, the bones were cropped in the transverse direction to remove the growth plate. Then, a sequence of morphological operators, including despeckling, closing and erosion, was applied to exclude the cortical bone layer^59^. The trabecular bone morphology was quantified with the software CTAn according to standard guidelines^56^, with the following parameters: bone volume fraction (BV/TV), trabecular thickness (Tb.Th), trabecular separation (Tb.Sp), and degree of anisotropy (DA). The latter was computed by fitting the mean intercept length (MIL) tensor with an ellipsoid and considering 1 minus the ratio between the smallest and the biggest eigenvalues, such that anisotropic and isotropic microstructures are defined by DA = 1 and DA = 0, respectively. The trabecular network was further characterized with a local spatially resolved analysis^60^, by defining cubic volumes of interest (VOI) marching along the cranio-caudal and the dorso-plantar directions (Fig. 2d). The size of each VOI was 0.75 mm (side length), so that about five trabeculae were included in each direction, to provide a spatially resolved and reliable estimation of trabecular architecture^56^. Starting from the most cranial region, the cube was displaced in steps of 0.375 mm (i.e., half of the side length) along the bone longitudinal axis of inertia and following the cranio-caudal direction (x-direction, Fig. 2d) until the growth plate. Seven locations were covered, for a total length of 3 mm. Spatial variations along the dorso-plantar direction (y-direction, Fig. 2d) were probed using marching cubes moving vertically, again in steps of 0.375 mm. Here, owing to the smaller dimensions of the calcaneus, only the most caudal locations were considered: two parallel sets of three regions could be analyzed, covering a length of 1.5 mm. Each cube contained only trabecular bone as ensured by simultaneous visualization of the three mutually perpendicular planes (Dataviewer, v.1.5.3.4, Skyscan). Within each VOI, the following local morphometric parameters were computed: BV/TV, Tb.Th, Tb.Sp, DA and preferred orientations (Pref.Or.θ and Pref.Or.ϕ). The latter were computed by considering the orientation of the MIL eigenvector with the smallest eigenvalue, which is an indication of the predominant spatial orientation of the trabeculae^61^. The two angles (Pref.Or.θ and Pref.Or.ϕ) were computed with respect to the principal axes of the bone^62,63^.

#### Bone microporosity: subchondral channels and fibrochondrocyte lacunae

**Figure 3:**
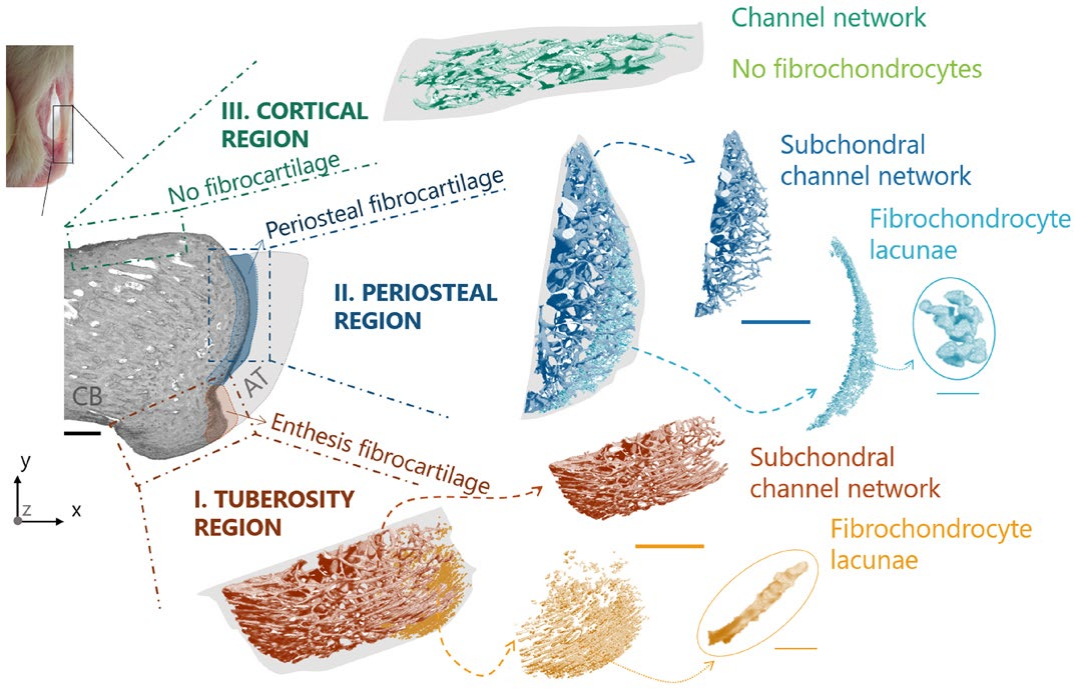
Representation of the three regions of interest defined for the analysis of bone microporosity along with three-dimensional renderings of the extracted subchondral channel network and fibrochondrocyte lacunae. The shaded gray area represents the considered tissue volume within each region. *CB* calcaneus bone, *AT* achilles tendon. Scale bars: 500 µm (bold), 50 µm (thin).

Bone microporosity was analyzed based on the high-resolution (i.e., 1.25 μm voxel size) scans and considering three distinct locations (Fig. 3): (*I*) the tuberosity region where tendon attaches to bone and encompassing the entire bony tuberosity and including mineralized (enthesis) fibrocartilage; (*II*) a periosteal region found on the caudal side of the calcaneus above the tuberosity and comprising subchondral bone covered with mineralized (periosteal) fibrocartilage; and (*III*) a cortical bone region situated beneath the bone surface away from the enthesis and lacking fibrocartilage. Those regions were identified in all samples based on approximately the same positions. The characterization of microporosity in subchondral bone and mineralized cartilage was conducted in CTAn (Fig. 3). The main contributor of microporosity in mineralized cartilage are fibrochondrocyte lacunae. In addition to (osteocyte) lacunae, the subchondral plate microporosity includes both vascular and avascular channels^64,65^. The former contain blood vessels while the latter are extensions of the marrow space and contain marrow cells and fat. As there is some overlapping in the dimensions of those features, a segmentation based on size is not feasible. Therefore, subchondral plate pores, other than lacunae, will be called subchondral channels^64^. Histology suggested that the majority of subchondral channels within the tuberosity and in the side region encloses blood vessels (“Results” section). The network of subchondral channels was extracted with the following procedure: a preliminary erosion operator (removing a layer of 125 μm in thickness) was applied to *Region I* and *II* to remove mineralized fibrocartilage. Then, a sweep method discarded all but the largest object. Bone porosity was highlighted by inverting the image, and then classified using a despeckle filter: objects with an area smaller than 20 pixels (i.e., corresponding to a diameter of about 6 µm in two-dimensional cross-sections), were considered to be either noise or osteocyte lacunae^46,66,67^. Finally, another sweep operator was applied to keep only the connected porosity, therefore ensuring to extract the subchondral channel network without interruptions. A similar algorithm was used to segment the fibrochondrocyte lacunae, now focusing on the layer of mineralized fibrocartilage discarded in the previous analysis. Here, instead of keeping the largest connected component, pores were thresholded and only objects between 1000 and 30,000 µm^3^ were considered to be fibrochondrocyte lacunae^68–70^. The upper threshold ensures that also large aggregates of fibrochondrocytes, which are a known feature of fibrocartilage^71^, were included in the analysis while excluding bigger objects (channels). Nevertheless, manual object removal was still necessary for some samples because of unconnected channel parts entering the region of interest. Renderings of the cell lacunae and channel network (Fig. 3; Supplementary Videos) were generated using CTVox (v.3.3.0, Skyscan) and CTVol (v.2.3.2.1, Skyscan).

The three-dimensional architecture of the channel network was quantified using both global and local morphometry. The global analysis was conducted in CTAn with a model-independent approach as done for trabecular bone^72^. The extracted global parameters included: total channel volume (Ch.V), channel volume density (Ch.V/TV), mean channel diameter (Ch.Dm), mean channel spacing (Ch.Sp), and degree of anisotropy (Ch.DA). The channel network was further characterized by a local analysis resolving individual channels. For that purpose, a Euclidean distance-ordered sequential homotopic thinning^73^ was applied to reduce the network to a one voxel thick skeleton without modifying the network topology. The voxels of the skeleton were then classified topologically as “branch” or “junction”^74^. The connected components of voxels marked as “junctions” were used as endpoints of the channels whereas the connected component of voxels classified as “branches” defined individual channels. All voxels belonging to an individual channel in the skeletonized image retained information on the corresponding Euclidean distance at that position, which can be interpreted as the local thickness of the channel in the original image. This measurement, along with channel connectivity, constituted the raw data used for the local channel morphometry. For each channel, the following local parameters were considered: channel length (Ch.L), channel diameter (Ch.Dm), channel connectivity (Ch.Conn), channel aspect ratio (Ch.ρ = Ch.Dm/Ch.L), and channel orientations (Ch.θ and Ch.ϕ). The two angles, polar (θ) and the azimuthal (ϕ), define a unit vector connecting the endpoints of a channel (Fig. 6). Since the channel network is an undirected graph structure, channel orientations were then mapped on an arbitrary half unit sphere by imposing that − 90° < θ < 90° and 0° < ϕ < 180°. Such constrains ensure that, for example, an angle of 10° is equivalent to an angle of 100° around the z-axis (Fig. 6). Channel skeletonization and processing were performed using ad hoc scripts developed in-house^75^ and integrated in Avizo.

The fibrochondrocyte lacunae and lacuna aggregates were characterized in CTAn using first global measurements, followed by a local analysis. Global parameters included total lacuna volume (L.V), lacuna volume density (L.V/TV), lacuna number (L.N) and lacuna number density (L.N/TV). Local three-dimensional measurements on individual lacunae comprised lacuna sphericity (L.Sph) and lacuna orientations (L.θ and L.ϕ). The sphericity was computed as the ratio of the surface area of an equivalent volume matched sphere to the surface area of the object (e.g., L.Sph = 1 indicates a perfectly spherical lacuna)^76^. Orientations were assessed by fitting each lacuna with an ellipsoid and by considering the orientation of the main axis of the ellipsoid with respect to the principal axes of the bone. Orientations were characterized by two angles in a spherical coordinate system—polar (θ) and the azimuthal (ϕ)—considering the same hemisphere and reference system used for the channels (Fig. 7).

#### Surface roughness

Surface roughness was measured on the high-resolution scans (1.25 μm voxel size) following a two-dimensional procedure^77,78^ implemented in Matlab. Three different regions of interest, previously defined for the analysis of bone microporosity, were considered (Fig. 7). Specifically, a rectangular mask (437.5 µm × 625 µm) was applied to extract the bone surface at (*I*) the interface between mineralized and unmineralized enthesis fibrocartilage located at the bony tuberosity; (*II*) the interface between mineralized and unmineralized periosteal fibrocartilage at the caudal side of the calcaneus; and (*III*) the bone surface (not covered by fibrocartilage) away from the enthesis and located on the dorsal side of the bone. For each sample, 10 sagittal cross-sections equally spaced about 30 μm apart, were studied for a total of 150 locations. In region (III), 6 masks (out of 50) were excluded because of the presence of blood vessels entering the bone (Supplementary Fig. S10). Surface profiles were defined based on contour voxels in the segmented micro-CT images. A mean reference surface was generated by approximating the extracted contour line with a 5th order polynomial. This choice ensures to account for the overall surface shape while avoiding the incorporation of local roughness caused for example by arrested fibrochondrocytes^79^. Surface height was computed as the shortest (Euclidean) distance from the reference line and roughness was measured as the root mean square deviation of the height profile from the mean line^78^. As the extraction of the bone contour is affected by the threshold, a sensitivity analysis was conducted to ensure that the main findings are robust against the specific value used to segment the bone (Supplementary Fig. S11).

### Statistics

Statistically significant differences in the morphological parameters of trabecular bone, in the global descriptors of the subchondral channel network and in the surface roughness, were investigated using a two-sample Student’s *t* test. Normality was checked by a Kolmogorov–Smirnov test, and variance using a two-sample F test. If one of those criteria was not met, a Mann–Whitney U test was employed. Significant differences in the three-dimensional orientation of channels and lacunae at the sites of interest were assessed with a non-parametric two-sample and two-dimensional Kolmogorov–Smirnov test, based on the Peacock algorithm^80^. This test determines whether two datasets are drawn from the same continuous distribution (without any assumption on the type of distribution). p values smaller than 0.05 were considered significant. The statistical analysis was done in Matlab (using the statistics toolbox and Central File Exchange)^81^.

## Results

### Histology and backscattered electron imaging

**Figure 4:**
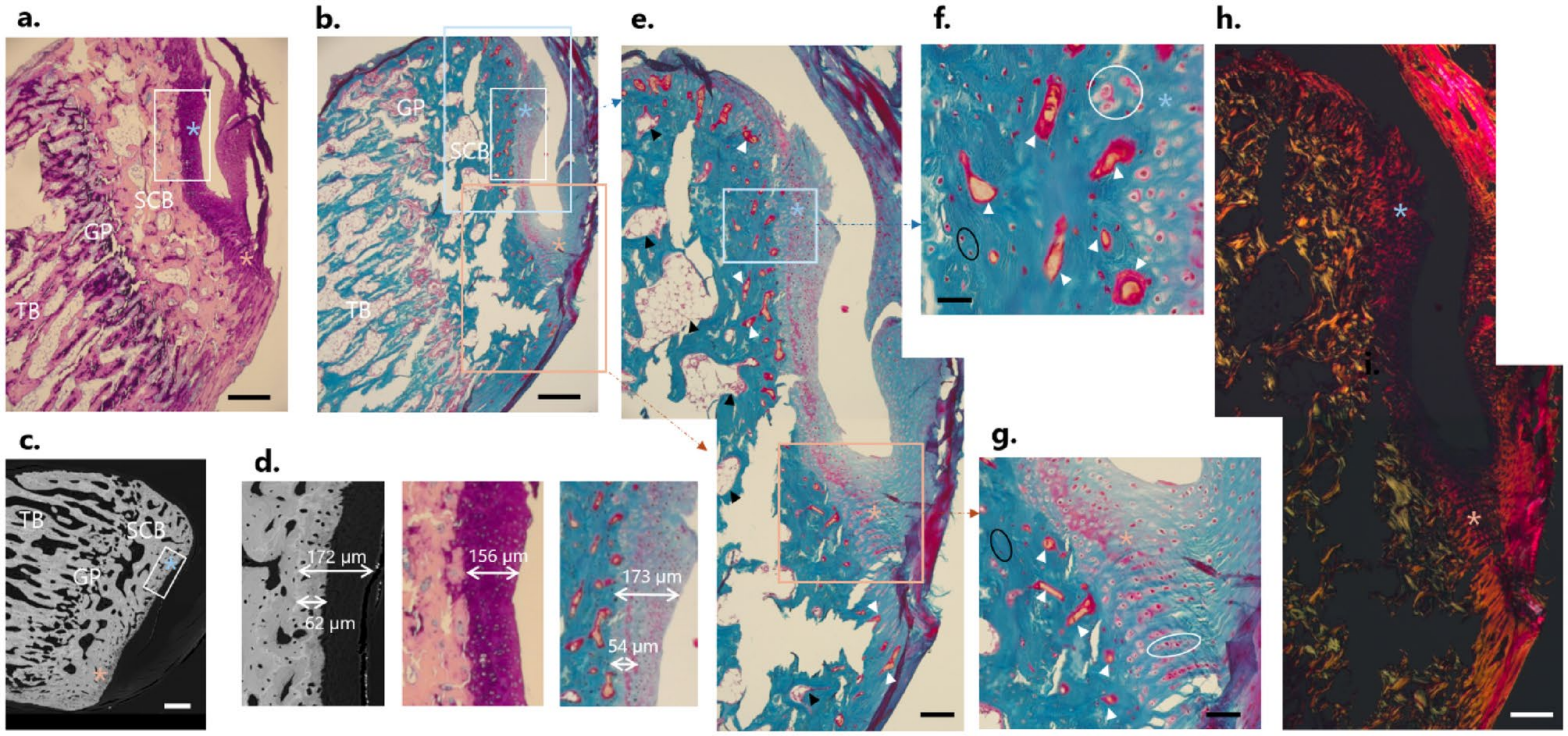
Histological and backscattered electron imaging (BEI) analysis from two sagittal sections stained with Giemsa and Goldner. Bright field light microscopy images of a thin section stained with Giemsa (**a**) and Goldner trichrome (**b**) at a similar location, as well as BEI (**c**), highlighting the areas of interest: trabecular bone (TB), growth plate (GP), subchondral bone (SCB), as well as periosteal fibrocartilage (light blue asterisk) and enthesis fibrocartilage (light pink asterisk). Scale bars: 250 µm. (**d**) Zoom from white frames in (**a**–**c**) on the periosteal fibrocartilage with BEI, Giemsa and Goldner, respectively, suggesting a thickness of the mineralized layer of about 50 µm corresponding to the reddish band in Goldner image. (**e**) Magnifications of light blue and pink frames from (**b**) illustrating some subchondral trabecular pores (black arrows) and subchondral vascular channels (white arrows). Scale bar: 100 µm. (**f**) Magnified view from frame in (**e**), revealing chondrocytes arrangement in fibrocartilage (white circle), as well as osteocytes in bone (black circle) and subchondral vascular channels (white arrows). Scale bar: 25 µm. (**g**) Magnified view at the insertion, from frame in (**e**), showing the column-like organization of chondrocytes, in close vicinity with osteocytes (black circle) and subchondral vascular channels (white arrow). Scale bar: 50 µm. (**h**) Polarized light microscopy corresponding to images in (e) suggesting a high degree of alignment of collagen fibers at the insertion. Scale bar: 100 µm.

As Giemsa stains proteoglycans, it allows to differentiate fibrocartilage from bone. In Fig. 4a, fibrocartilage appears in dark violet and bone in light pink. This staining highlighted the locations of periosteal (light blue asterisk) and enthesis (light pink asterisk) fibrocartilages on the caudal portion of the calcaneus. The histological analysis also revealed an intricate pattern of interdigitations at the insertion region, with fingers of fibrocartilage deeply inserting into subchondral bone. The periosteal region, in comparison, showed a smoother interface between fibrocartilage and bone. The growth plate zone could also be identified thanks to the presence of several cartilage inclusions. Likewise, cartilage islands were also found in other locations away from the growth plate, and they are a well-known feature of unremodeled rat bone^82^. Figure 4b shows an overview of a Goldner stained section at a similar location. This stain colors bone mineralized matrix in green, non-mineralized tissue, such as osteoid, in red, and erythrocytes in bright orange. Figure 4c shows a cross section analyzed with BEI at a similar location, enabling to distinguish mineralized and unmineralized areas. A magnified view in Fig. 4e highlights the high heterogeneity of the insertion region. Avascular subchondral trabecular pores containing marrow space (black arrows) could be observed. Smaller cavities were also detected close to the fibrocartilage layer, probably filled with osteoid and erythrocytes (white arrows). Both avascular and vascular channels are known features of the subchondral bone plate and mineralized cartilage^64^. A reddish band, approximately 50 µm away from bone and corresponding to the end of the mineralized area in BEI, demarcates mineralized from non-mineralized cartilage, as is illustrated in Fig. 4d. Figure 4f,g present a magnified view on the cell population at the fibrocartilage insertions. First, the presence of fibrocartilage is confirmed by the observation of roundish fibrochondrocytes at both sites (white circles), yet of different arrangement. At the enthesis, they formed columns while the periosteal chondrocytes did not show specific organization. Osteocytes, much smaller and less round than fibrochondrocytes, could also be spotted at the bone side (black circles). In Fig. 4h, a polarized light microscopy image of the entire caudal portion of the calcaneus (including enthesis and periosteal region) is reported. A qualitative examination revealed a bright signal at the insertion (light pink asterisk) while the periosteal area remained dark (light blue asterisk), suggesting a different alignment of the collagen fibers in the two regions. Fig. S4 provides additional similar histological images performed on a different sample.

### Tuberosity

The Achilles tendon inserts into the caudal portion of the calcaneus at a marked bony tuberosity, comprising not only mineralized fibrocartilage but also a large portion of subchondral bone, with a total tissue volume T.TV of 0.57 mm^3^ ± 0.05 mm^3^. The tuberosity had quite a low porosity (T.Po = 14.84% ± 2.16%, measured at 1.25 μm voxel size) indicating that plate-like subchondral cortical bone prevailed over subchondral trabecular bone^83,84^. The protrusion also had a clear global orientation with respect to the inertia axes of the calcaneus, being fairly well aligned along the sagittal plane, as implied by the polar angle T.θ of 94.79° ± 1.5°, and pointing upwards in the direction of the tendon, with an inclination (azimuthal angle T.ϕ) of 21.26° ± 3.57° (Fig. 2c).

### Trabecular morphology and spatially resolved analysis

The trabecular bone in the caudal region of the rat calcaneus was rather dense (BV/TV = 62.08% ± 3.3%) with the trabeculae being not particularly thick (Tb.Th = 103.5 µm ± 4.87 µm) but quite packed (Tb.Sp = 98.7 µm ± 9.15 µm). Overall, the trabecular network was also fairly anisotropic (DA = 0.48 ± 0.05). Those values are in line with previous studies on rat trabecular bone^83^.

**Figure 5:**
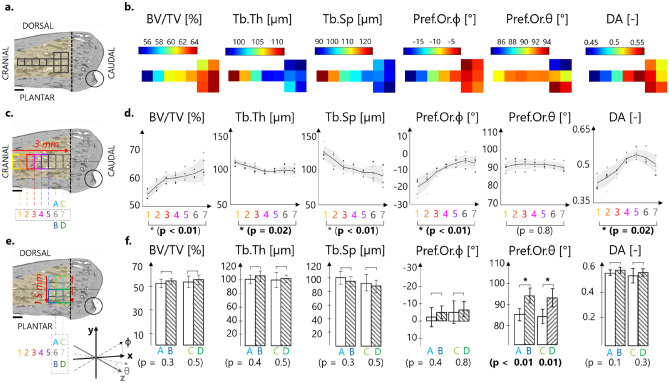
Three-dimensional spatially resolved analysis of trabecular bone architecture (the analyzed trabecular compartment is highlighted in yellow). (**a**) Overview of the central part of the regions of interest (black squares), with the analyzed parameters color-coded in (B). (**b**) Spatial evolution of bone microstructural parameters along the cranio-caudal (CC) and the dorso-plantar (DP) directions. (**c**) Illustration of the volumes of interest (VOI) for the CC investigation at 7 consecutive positions (i.e., 1, …, 7). (**d**) Spatial evolution of morphometric parameters along the CC direction showing significant variations in BV/TV, Tb.Sp and Pref.Or.ϕ when comparing the two extremities (i.e., positions 1 and 7). (**e**) Illustration of the VOI for the DP investigation considering 2 dorsal (A and C) and 2 plantar (B and D) locations. (**f**) Comparison of trabecular parameters averaged within dorsal and plantar regions revealing significant changes only for Pref.Or.θ. Scale bars: 500 µm. Statistically significant p values (p < 0.05) are shown in bold.

Local spatial variations in trabecular microarchitecture were investigated to assess whether bone microstructure away from the highlighted tuberosity shows distinct patterns, which could be related to tendon attachment. The architectural descriptors were characterized along the cranio-caudal (CC) and the dorso-plantar (DP) directions (Fig. 5a,b). Quantitative assessment of trabecular microstructure along the cranio-caudal direction highlighted significant variations (p < 0.05) when approaching the growth plate for some morphological parameters (Fig. 5c,d). For example, BV/TV increased from 54.5 to 64.4% with a slope of about 4%/mm, mostly due to a decrease in trabecular spacing, with trabecular thickness being practically constant. The local predominant azimuthal orientation (Pref.Or.ϕ) of the trabecular network changed from about − 19.5° to − 5° when going closer to the growth plate, meaning that the trabeculae pointed initially towards the tuberosity and then rotated to align along with the cranio-caudal direction. The polar angle Pref.Or.θ did not show significant variations and stayed around 90°, suggesting that the trabecular network was parallel to the sagittal plane. Changes in trabecular orientation were accompanied by a significant increase of about 20% in the degree of anisotropy (from 0.44 to 0.52, p < 0.05). Despite the presence of the tendon insertion and related tuberosity, almost none of the trabecular parameters showed significant differences when comparing dorsal versus plantar locations (Fig. 5e,f). Only the polar angle (Pref.Or.θ) significantly increased, indicating that plantar trabeculae were slightly more co-aligned with the sagittal plane.

### Subchondral channel network

Although belonging to the same bone and being adjacent to each other, the three regions introduced to study bone microporosity (Fig. 3; Supplementary Videos S1, S2, S3) showed large differences in the subchondral channel network, as highlighted by the global morphological analysis (Table 1). Subchondral bone beneath periosteal fibrocartilage (*Region II*) had the highest Ch.V and Ch.V/TV, followed by the tuberosity (*Region I*) and cortical bone (*Region III*). In the latter, Ch.V/TV was about 42% smaller than in the tuberosity (p < 0.01). The high channel density in the two regions beneath fibrocartilage (i.e. *Region I* and *II*) was due to fewer but larger and more separated channels in comparison with cortical bone. Interestingly, the three regions had strong dissimilarities in the degree of anisotropy, with the insertion site (*Region I*) showing Ch.DA more than a factor of two higher than at the periosteal site (*Region II*), and only 26% smaller than within cortical bone (*Region III*).

**Table 1:** Global morphometric parameters used to characterize bone microporosity.

Parameter	Tuberosity (*Region I*)	*p* (I–II)	Periosteal (*Region II*)	*p* (II–III)	Cortical (*Region III*)	*p* (I–III)
**Subchondral channel parameters**						
Ch.V (mm^3^)	0.025 ± 0.01	**0.02**	0.05 ± 0.02	** < 0.01**	0.01 ± 0.003	** < 0.01**
Ch.V/TV (%)	6.21 ± 1.72	**0.02**	10.80 ± 2.22	** < 0.01**	3.84 ± 0.80	** < 0.01**
Ch.Dm (µm)	36.85 ± 15.92	0.15	50.36 ± 3.88	**0.02**	21.87 ± 2.69	** < 0.01**
Ch.Sp (µm)	100.51 ± 4.84	0.09	106.25 ± 2.16	**0.02**	121.83 ± 3.95	**0.03**
Ch.DA (–)	0.56 ± 0.03	** < 0.01**	0.21 ± 0.06	** < 0.01**	0.71 ± 0.02	** < 0.01**
**Fibrochondrocyte lacuna parameters**						
L.V (mm^3^)	0.005 ± 0.0006	** < 0.01**	0.0078 ± 0.001	–		
L.V/TV (%)	2.24 ± 0.4	0.2	2.86 ± 0.7	–		
L.N (–)	1415 ± 256.6	** < 0.01**	2555 ± 230.6	–		
L.N/TV (/mm^3^)	274,845 ± 38,719	0.056	329,447 ± 22,090	–		

Values are mean ± standard deviation. Statistically significant p values (p < 0.05) are shown in bold.

*Ch.V* total channel volume, *Ch.V/TV* channel volume density, *Ch.Dm* mean channel diameter, *Ch.Sp* mean channel separation, *Ch.DA* channel degree of anisotropy, *L.V* total lacuna volume, *L.V/TV* lacuna volume density, *L.N* lacuna number, *L.N/TV* lacuna number density.

**Figure 6:**
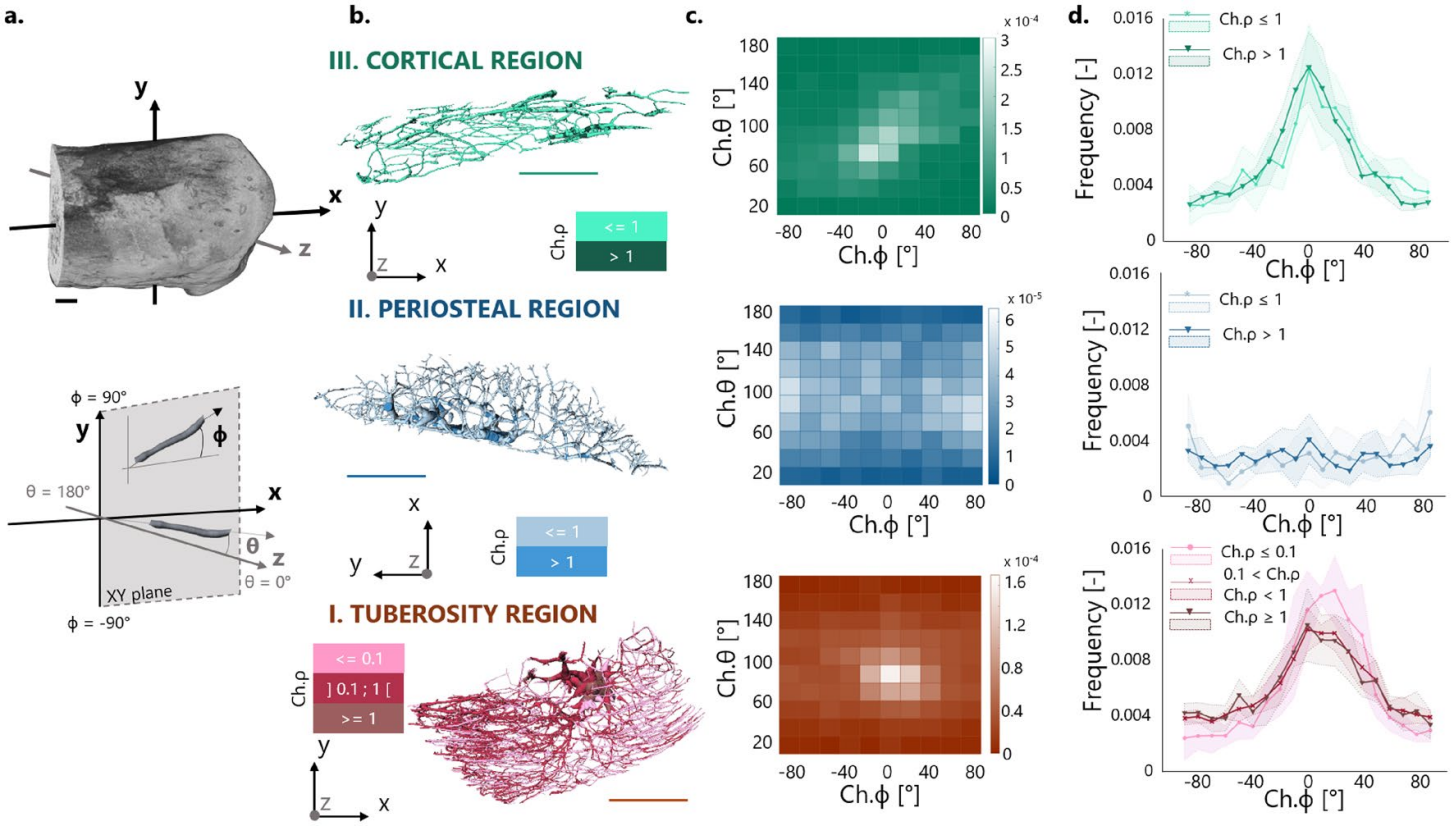
Three-dimensional local analysis of the subchondral channel network at the three sites of interest based on the high-resolution micro-CT scans. (**a**) Representation of the spherical coordinate system with the azimuthal (ϕ) and polar (θ) angle defining the orientation of the channels. (**b**) Illustration of extracted channel network skeletonized to resolve individual channels, which are classified and colored according to the aspect ratio as thick, slender and extremely slender. (**c**) Representative two-dimensional heat maps of channel orientation, revealing a specific pattern for each region. Dataset relative to one animal and data normalized to unit volume. (**d**) Frequency distributions (normalized to unit area) of the azimuthal angle (Ch.ϕ) describing the orientation of the channel in the XY sagittal plane for the entire dataset. Channels are classified according to the aspect ratio as thick, slender and extremely slender. Data reported as mean value (thick lines) with standard deviation (shaded areas). Scale bars: 500 µm.

To gain more insight in the arrangement of the channel network, a local analysis resolving individual channels was performed (Fig. 6). By knowing the local orientation of each channel (expressed as two angles Ch.θ and Ch.ϕ, Fig. 6a,b), two-dimensional heat maps of the channel orientation were obtained for each region of interest (Fig. 6c; Supplementary Fig. S7). The maps, generated by binning channel angles within a 20° interval, highlighted a strong anisotropy for the tuberosity and the cortical regions, in striking difference with respect to the more isotropic subchondral bone beneath periosteal fibrocartilage. In particular, channels within the tuberosity had a preferred orientation (corresponding to the maximum in the heat map) characterized by Ch.ϕ = 10.6° ± 8.6° and Ch.θ = 81.8° ± 3°. A polar angle Ch.θ close to 90° indicates that channels were rather parallel to the sagittal plane (XY plane in Fig. 6), yet somewhat oriented towards the lateral side of the calcaneus. The positive azimuthal angle Ch.ϕ underlines that channels pointed upwards (dorsally) in the direction of the tendon. The three regions had heat maps significantly different from each other, as indicated by a two-dimensional Kolmogorov–Smirnov test (p < 0.01, Supplementary Table S1). The channels were further classified, according to their aspect ratio Ch.ρ, into three groups: thick (Ch.ρ ≥ 1), slender (0.1 < Ch.ρ < 1) and extremely slender (Ch.ρ ≤ 0.1) channels. Frequency distributions of Ch.ϕ were obtained in the different regions and for the three groups (Fig. 6d). In the tuberosity, all distributions showed a clear peak but different peak positions and widths. Considering extremely slender channels, located predominantly on the Achilles tendon insertion side of the tuberosity (Fig. 6b), the most frequent azimuthal orientation was 45% higher than that of less slender channels, indicating an increased tilt pointing towards the attachment region. Additionally, they also showed a somewhat reduced orientation heterogeneity as indicated by a narrower peak. In the cortical bone region, the frequency distributions peaked at Ch.ϕ = 2° both for thick and slender channels, indicating a preferential longitudinal arrangement (i.e. along the cranio-caudal axis), independently of the channel aspect ratio. A clearly different behavior was observed within subchondral bone beneath periosteal fibrocartilage, where channels had a fairly random (isotropic) orientation. Furthermore, frequency distributions of channel length, diameter and connectivity in the different regions showed similar bell-shaped patterns with channels in the tuberosity having the tendency to be thinner and more branched in comparison with the cortical region (Supplementary Fig. S5). One additional distinct feature of the tuberosity common to all analyzed bones was the presence of a rather big central channel branching into smaller channels at an almost right angle when reaching the tuberosity (Supplementary Fig. S6).

### Fibrochondrocyte lacunae

**Figure 7:**
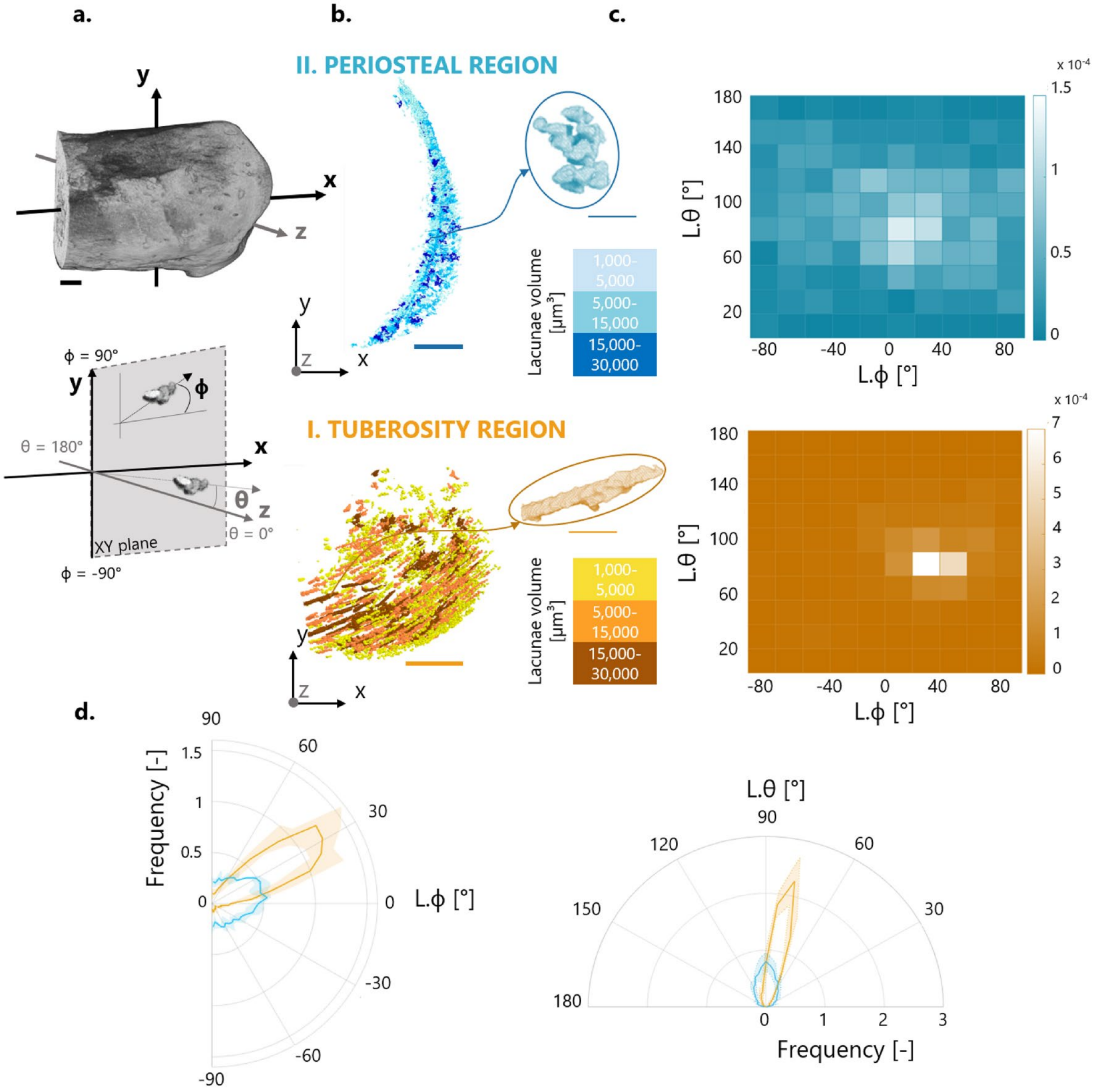
Three-dimensional local analysis of the fibrochondrocyte lacunae within the two locations of interest and based on the high-resolution micro-CT scans. (**a**) Representation of the spherical coordinate system with the azimuthal (ϕ) and polar (θ) angles defining the orientation of the lacunar porosity. (**b**) Illustration of extracted lacuna aggregates for the two regions of interest. Lacunae are classified and colored according to their volume as small, intermediate and big. (**c**) Representative two-dimensional heat maps of lacuna orientation, revealing a clear preferred orientation at the insertion. Dataset relative to one animal and data normalized to unit volume. (**d**) Circular plots of the azimuthal angle (describing the orientation of the pores in the XY sagittal plane) and of the polar angle (depicting the orientation with respect to the z-axis) for the entire dataset. Data reported as mean value (thick lines) with standard deviation (shaded areas). Plots are normalized to unit area. Scale bars: 500 µm (thick) and 50 µm (thin).

Three-dimensional visualization of lacunar porosity (Fig. 7) revealed that fibrochondrocyte lacunae formed specific aggregates depending on the considered location (Fig. 7b; Supplementary Videos S4, S5): at the mineralized enthesis fibrocartilage covering the most caudal part of the tuberosity, lacunae piled up to form elongated columnar structures, whereas at the mineralized periosteal fibrocartilage bordering subchondral bone, lacunae aggregated into three-dimensional clusters. Global morphometric parameters (Table 1) indicated that the two mineralized fibrocartilages had similar lacuna volume density and lacuna number density. At the individual level, pore sphericity was negatively correlated with pore volume, suggesting that small lacunae were consistently the most spherical ones (Supplementary Fig. S8). To target the specific arrangement of lacunar aggregates, the orientation analysis was restricted to pores of low sphericity (i.e., L.Sph < 0.6). Two-dimensional heat maps of lacuna orientation (L.θ and L.ϕ) showed strong and statistically significant differences (p < 0.01, Supplementary Table S2) between the two regions (Fig. 7c; Supplementary Fig. S9). Fibrochondrocyte lacunae forming columns located at the tuberosity were well oriented at an azimuthal angle of L.ϕ = 31.1° ± 4.4° (Fig. 7d), indicating that the columns pointed towards the tendon insertion site with a characteristic inclination. Rows corresponding to the biggest aggregates seemed to be located on the plantar side of the tuberosity (Fig. 7b). In the mineralized periosteal fibrocartilage, lacunar orientation was much less anisotropic, with azimuthal angles in the range 0° to 30° being almost equally likely. As for the channels, the polar angle L.θ within enthesis fibrocartilage was close to 80° (L.θ = 79.1° ± 1.8°), highlighting a somewhat lateral arrangement of the lacuna columns and likely reflecting the predominant polar orientations of the tuberosity (Fig. 7d).

### Roughness

**Figure 8:**
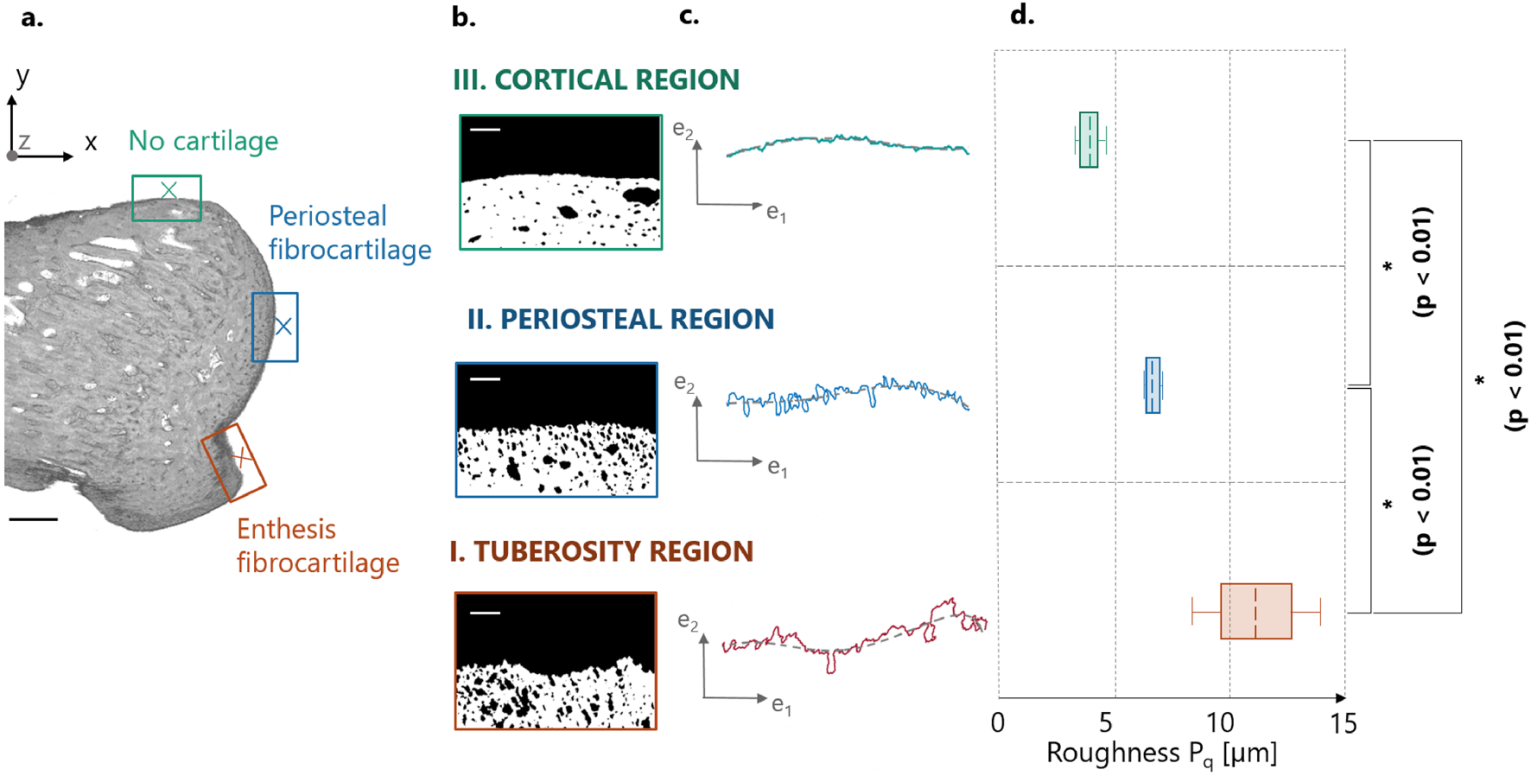
Roughness of the interface profile line based on the high-resolution micro-CT scans. (**a**) Regions of interest (ROI) on a representative bone cross section. (**b**) ROI after extraction and binarization. (**c**) The full line represents surface profile whereas the dashed line shows the reference line used to compute the roughness. e_1_ and e_2_ are local direction vectors. (**d**) Surface roughness (expressed as root mean square P_q_) calculated in the three ROI including the interface between mineralized and unmineralized enthesis fibrocartilage (I. Tuberosity region), the interface between mineralized and unmineralized periosteal fibrocartilage (II. Periosteal region) and the outer cortical bone surface (III. Cortical region). Box plots extends from 25 to 75th percentiles and whisker bars covers the full data range. The dashed line represents the mean value. Scale bars: 500 µm (in A, black), 100 µm (in B, white). Statistically significant p values (p < 0.05) are shown in bold.

Roughness was significantly different (p < 0.01) among the three analyzed surfaces (Fig. 8). Mean roughness (expressed as root mean square P_q_) at the interface between mineralized and unmineralized enthesis fibrocartilage (present at the tuberosity) was 65% higher than at the interface between mineralized and unmineralized periosteal fibrocartilage (covering subchondral bone), and 172% higher than at the outer cortical bone surface. The latter was also significantly less rough (about − 39%) than the regions of bone covered by mineralized periosteal fibrocartilage. Those results were robust against the threshold value considered for roughness computation (Supplementary Fig. S11).

## Discussion

In this work, we have analyzed microstructure and microporosity of bone and mineralized fibrocartilage at a clinically relevant location, where the Achilles tendon is attached to the calcaneus bone in rats. This region is highly heterogeneous and features different mineralized tissues such as the thin and irregular mineralized fibrocartilage layer, the dense subchondral plate, the porous subchondral trabecular bone and, after the growth plate, the trabecular compartment. To highlight different functional aspects of those regions, we have characterized either the microstructure (Figs. 5, 8) or the microporosity (Figs. 6, 7). Specifically, dense tissues such as the mineralized fibrocartilage and the subchondral plate were analyzed in terms of microporosity whereas the highly porous trabecular bone region and the irregular interface between mineralized and non-mineralized fibrocartilage were characterized in terms of microstructure. Although, in humans, this region suffers from overuse injuries, rheumatic pathologies and fractures, it has been much less investigated than tendon or bone^3^. In addition to the clinical relevance, the calcaneus bone presents attractive features for investigating mechanobiological questions. Firstly, it solves the clear biomechanical task of transmitting the tendon force to the ankle joint and, in analogy with a cantilever beam, the dorsal side is prevalently loaded in compression while the plantar side is loaded in tension^85^. In case of off-axis loading, shear strains may also be present^86^. Due to this fairly simple loading condition, the calcaneus has been used to characterize material and architectural adaptation to mechanical forces in different species^86–89^. Moreover, the bone receives the load from the tendon at a well-localized region, thus offering the additional opportunity to investigate local adaptation strategies of the mineralized tissues close to the insertion site.

Here, we have found that the calcaneus bone of rats presents a dedicated protrusion, referred to as tuberosity, to anchor (part of) the tendon. The existence of a specialized bony region at the enthesis is common to other bones^90^, and plays a role in joint functioning. For example, at the Achilles tendon insertion in humans, the caudal tuberosity of the calcaneus is assumed to increase the lever arm of the tendon, thus providing a biomechanical advantage^3^. The tuberosity is also a possible indicator of muscle activity: from a mechanobiological point of view, a pronounced tuberosity may reflect increased muscle loading. For this reason, bony tuberosities have been investigated in the archeological context to learn about physical activities of past populations^91^. Noteworthy, tendon fibers attaching at the tuberosity of calcaneus bone (in mice) have a different arrangement than the surrounding fibers. This suggests that the protrusion could experience a different loading condition than nearby bone^6^. To further investigate the role of the tuberosity in load transfer, we have performed a spatially resolved analysis of the calcaneus trabecular microstructure, which revealed gradients when moving towards the growth plate along the cranio-caudal direction but not when approaching the tuberosity along the dorso-plantar direction. The presence of microstructural gradients in the trabecular network -especially in porosity- may serve to mitigate stress incompatibilities when transitioning from the more compliant trabecular region to the stiff cortical bone^92^. This is a known feature of long bones^60,93^. However, the lack of significant changes in trabecular architecture along the dorso-plantar direction, despite moving closer to the tendon insertion, suggests that the tuberosity may facilitate the “force flow” from the Achilles tendon to the plantar fascia ligament, somewhat relieving the trabecular network from such task. Additional mechanical analysis, for example based on microstructural finite element simulations^94,95^, should be performed to confirm this assumption.

The caudal calcaneus features three types of fibrocartilage, of which two are contiguous but with specific biomechanical functions and developmental origin: enthesis fibrocartilage, anchoring tendon to bone, originates from the cartilage rudiment of the calcaneus and has analogies with the growth plate that is formed during endochondral ossification^90^, whereas periosteal fibrocartilage, facilitating tendon sliding and protecting bone, arises from the calcaneal perichondrium^51,71^. Owing to their tasks, the two tissues are subjected to distinct loading conditions. Enthesis fibrocartilage should prevalently accommodate the large tensile force of the tendon, with local compressive and shear strains emerging at the interface^5,37,96^. The periosteal fibrocartilage should sustain overall compressive and shear loading^71^, likely of smaller magnitude than enthesis fibrocartilage. By comparing the mineralized regions of enthesis and periosteal fibrocartilage, we have found large dissimilarities in surface roughness and microstructural porosity. Firstly, the roughness of the interface between mineralized and unmineralized tissues, caused by the mineralization front and by the arrested fibrochondrocytes, is the highest at the tuberosity. From a mechanical point of view, introducing surface pattering in the form of random roughness or controlled interdigitations is a well-known strategy to increase the fracture resistance of bi-material junctions^9,97,98^. At the tendon-bone attachment there are actually two interfaces of interest: the transition between unmineralized and mineralized fibrocartilage, referred to as the tidemark or mineralization front, and the one gluing mineralized fibrocartilage to bone (often featuring a thin interlayer, called cement line). Clearly, the roughness characterized in this work is at the mineralization front, yet both interfaces are far from being flat: the interface between mineralized fibrocartilage and bone probably shows the highest roughness caused by deep interdigitations between the two mineralized tissues^99,100^. Interface waviness is a general feature of the attachment between bone and soft tissues, including tendon, ligament, cartilage and meniscus^6,11,13,101^. At the tendon insertion, roughness is well conserved across different species but does not seem to increase much with animal size and muscle loading, perhaps because higher loading is often accompanied by a larger attachment area, resulting in a fairly constant interfacial stress with no requirement to adapt surface properties^12^. Nevertheless, we observed significant differences when comparing roughness between enthesis and periosteal location, possibly because the former has to sustain higher stresses. Others have also found differences in roughness between the two bundles of the ACL attaching to the tibia, which may reflect site-specific biomechanical requirements^99^. The roughness profiles measured here may be combined with computational models^9,102^ to estimate their impact on interface strength and fracture behavior^9^.

In the two mineralized fibrocartilages, we also found specific arrangements of fibrochondrocyte lacunae, which formed clusters having distinct shapes and orientations. Similar to osteocytes, fibrochondrocytes live buried in the challenging environment of the mineralized matrix. In bone, the arrangement of the osteocytes is not fully random but reflects the three-dimensional organization of the collagen matrix^103^ and is influenced by the local mechanical environment^47,104,105^ as well as by systemic factors such as aging^106^ and diseases^107^. Although fibrocartilage does not have the same remodeling ability than bone, the same considerations may hold (at least partially) for the fibrochondrocyte lacunae. Mechanical loading has been shown to be necessary for the growth and maturation of the enthesis^108–110^, but also plays a critical role for interface healing^109^. During development, the whole process of chondrocyte proliferation, hypertrophy and bone formation is tightly regulated by biological signals and influenced by applied load^110,111^. For example, a specific population of cells probably linked to the regulation of enthesis development, has been identified at the fibrocartilaginous enthesis as early as in perinatal period. Some hallmarks of these cells seem to be influenced by muscle loading^110^. From a mechanobiological point of view, enthesis development and healing are intimately connected with local mechanical cues. Likewise, the organization of mature tissues at enthesis may mirror the local mechanical environment. At the attachment region, although varying in orientation with gait cycle and motion, a rather clear and localized predominant loading condition is provided by the tendon, and this is where the lacunae exhibit the highest alignment, also as a possible way to minimize dangerous stress localization around those large pores. This may also be the case for the highly aligned subchondral channels. Looking outside the mammalian musculoskeletal system, chondrocytes within mineralized cartilage of rays also show pronounced spatial organization and orientation, perhaps reflecting differences in the local loading environment^70^. The spatial organization of fibrocartilage cells may mirror the arrangement of the underlying collagen network. Indeed, in enthesis fibrocartilage, collagen fibrils assemble into parallel bundles with the cells forming elongated column-like structures sandwiched in between those bundles; conversely, in periosteal fibrocartilage, collagen exhibits a more disordered and interwoven network and cells tend to be more rounded and less ordered^51,71,112,113^.

In addition to cell lacunae, we have also shown that the channel network inside the tuberosity is strongly anisotropic: channels were fairly well oriented along a predominant direction pointing towards the attachment region and differing from the disposition of a control cortical bone location, as well as from the organization of channels at the periosteal location, which were more randomly arranged. Bone adaptation to mechanical loading has been extensively investigated at the organ and tissue level, but much less at the level of the subchondral channel network. However, there is evidence that loading affects channel orientation even in primary cortical bone as immobilized bones show more radially oriented channels than loaded bones^46^. Here, we have highlighted that tendon loading may as well have an impact on the three-dimensional organization of the channel network. Finally, collagen fiber alignment has been shown to regulate (in vitro) the formation and alignment of the vascular network^114^. These mechanisms may play a role at the tuberosity as well. That being said, it should be kept in mind that entheses are known to be highly heterogeneous and anatomical site-dependent tissues, mainly because of specific and varying loading environments: for example, the anterior cruciate ligament shows structural and mechanical differences when comparing its femoral and the tibial insertions^13^.

Turning to the limitations of this work, we have analyzed a limited number of samples. However, the central aim of our study was not to perform a comparison between groups, which would have required a larger number of animals, but to investigate specific locations within the same bone, likely undergoing dissimilar loading conditions. As we considered bones of laboratory rodents, all having the same age and raised in the same environment, the biological variability was strongly limited. Moreover, we characterized micrometer level features and the considered set of samples allowed extracting and analyzing about 46,000 channels and 20,000 lacunae, obtaining results which were not only statistically significant but also had fairly low standard deviations (as visually evidenced by the Supplementary Videos). In an effort to reduce the number of animals exploited, many studies aiming at comparing several sites within the same bone rather than intergroup comparisons, are based on a limited number of samples^6,60,70,115^. Besides the similarities between humans and rodents in terms of bone architecture^116^, ankle anatomy and physiology, the main advantage of analyzing murine calcanei is their small size, allowing to measure at high-resolution (around 1 µm) a large bone region comprising the entire tuberosity, subchondral bone and mineralized fibrocartilages (enthesis and periosteal). However, it should be noted that our analysis does not allow distinguishing lacunae closer than the micro-CT resolution. While the grouping of fibrochondrocytes at the tendon insertion has been well documented^71^, the existence of aggregates within periosteal fibrocartilage has been less explored. Nevertheless, considering the limited communication possibilities among fibrochondrocytes within the mineralized matrix, having cells very close to each other may facilitate the exchange of information. Finally, the enthesis is a complex multi-tissue system and in this work, we have focused on the mineralized regions as they display a clear microstructural porosity which can be connected with the loading environment. Other complementary aspects of the unmineralized region of the enthesis may be investigated using, for example, contrast enhanced micro-CT^6^.

In conclusion, this study has demonstrated that close to the tendon insertion site, bone as well as mineralized fibrocartilage exhibit specific microstructural features, including a strong anisotropy and interdigitations which should be considered when designing future tissue engineering replicates of the bone-tendon system or in the context of novel re-attachment strategies. Future studies, considering for instance aged or diseased animals, could exploit our approach to investigate how microstructural aspects are impacted by tissue degeneration, addressing the complex and poorly understood interplay between bone and fibrocartilage in enthesis related pathologies.

## Supplementary Information

Supplementary Video 1.

Supplementary Video 2.

Supplementary Video 3.

Supplementary Video 4.

Supplementary Video 5.

Supplementary Information 1.

Supplementary Information 2.
