# Supplementary material for: Local anisotropy in mineralized fibrocartilage and subchondral bone beneath the tendon-bone interface
Source: Sci Rep. 2021 Aug 16;11:16534. doi: 10.1038/s41598-021-95917-4 (PMC8367976; doi:10.1038/s41598-021-95917-4)
Supplement: Supplementary file 6 — Supplementary Information 1. [file 41598_2021_95917_MOESM6_ESM.pdf]

Supplementary material (Video Captions)

Video S1. Three-dimensional visualization of the subchondral channel network for the tuberosity site of interest based on the high-resolution micro-CT scans. Scale bar: 250 μm.

Video S2. Three-dimensional visualization of the subchondral channel network for the subchondral site of interest based on the high-resolution micro-CT scans. Scale bar: 250 μm.

Video S3. Three-dimensional visualization of the subchondral channel network for the cortical site of interest based on the high-resolution micro-CT scans. Scale bar: 250 μm.

Video S4. Three-dimensional visualization of the fibrochondrocyte lacunae at the tuberosity site of interest based on the high-resolution micro-CT scans. Scale bar: 250 μm.

Video S5. Three-dimensional visualization of the fibrochondrocyte lacunae at the subchondral site of interest based on the high-resolution micro-CT scans. Scale bar: 250 μm.
