# Supplementary material for: Local anisotropy in mineralized fibrocartilage and subchondral bone beneath the tendon-bone interface
Source: Sci Rep. 2021 Aug 16;11:16534. doi: 10.1038/s41598-021-95917-4 (PMC8367976; doi:10.1038/s41598-021-95917-4)
Supplement: Supplementary file 7 — Supplementary Information 2. [file 41598_2021_95917_MOESM7_ESM.pdf]

Local anisotropy in mineralized fibrocartilage and subchondral bone beneath the tendon-bone interface

by Alexandra Tits, Erwan Plougonven, Stéphane Blouin, Markus A. Hartmann, Jean-François Kaux, Pierre Drion, Justin Fernandez, G. Harry van Lenthe, Davide Ruffoni

Supplementary material (Figures & Tables)

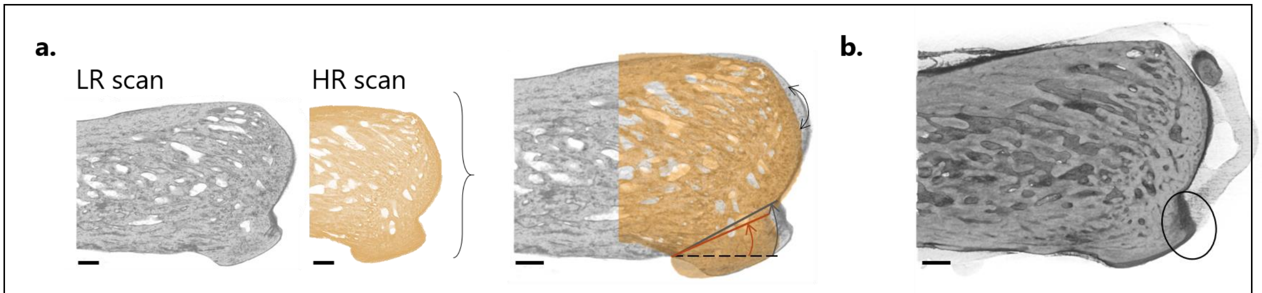

Fig. S1. Image alignment and tendon visualization. (**a**) Co-alignment procedure between the low resolution (LR, 5 μm voxel size) and the high resolution (HR, 1.25 μm voxel size) scans based on the orientation of the tuberosity. (**b**) Visualization of the Achilles tendon inserting into the bone at the tuberosity, obtained measuring bone-tendon samples in air (without embedding) and after a manual threshold highlighting the soft tissues. Scale bars: 500 μm.

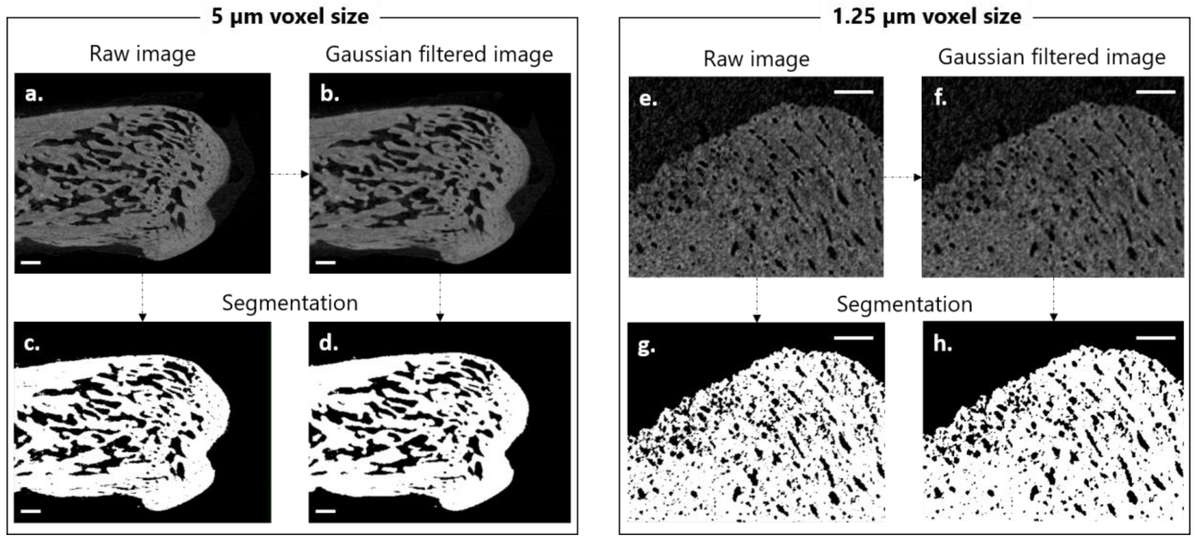

Fig. S2. Gaussian filtering and binarization on representative cross-sections of the 5 µm and 1.25 µm scans. Filtering allows to remove image noise causing dot-like artifacts in the binarized data. As those tiny features were not considered in our analysis, and as we did relative comparisons among different regions of the same bone, we expect filtering not to impact our main findings. Scale bars: (a-d) 500 µm; (e-h) 100 µm.

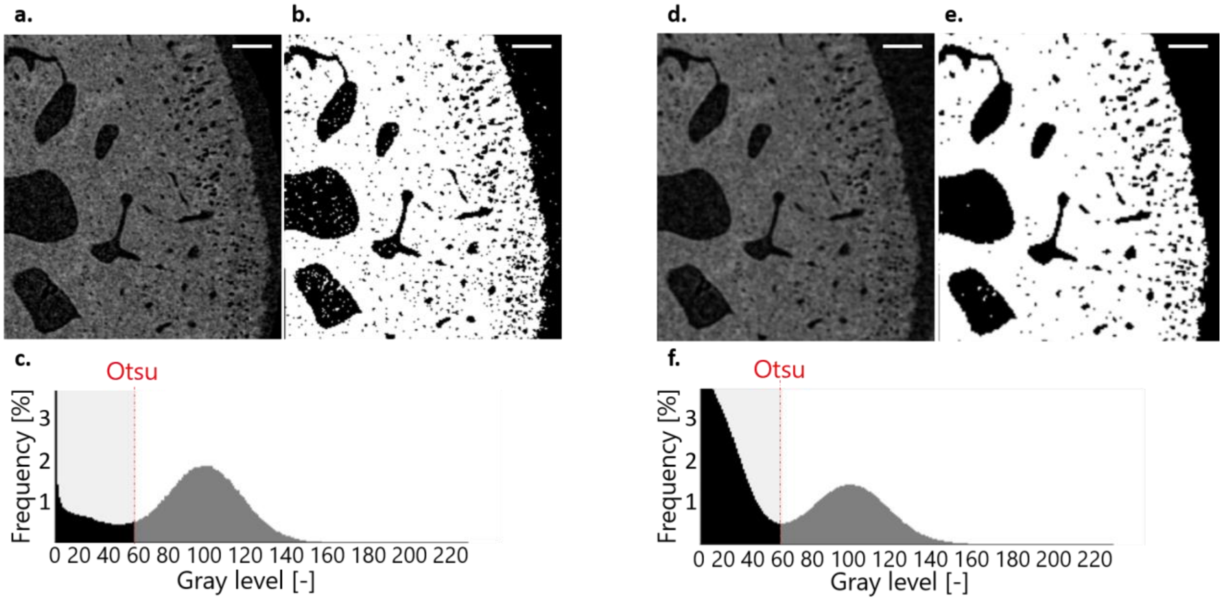

Fig. S3. Illustration of Otsu's threshold on the original cross-section (a-c) and after filtering (d-f). Filter enhances the histogram bimodality, ensuring the efficiency of Otsu's algorithm to segment foreground from background voxels [58]. Scale bars: 100 µm.

Table S1

Results of the two-sample two-dimensional Kolmogorov-Smirnov tests comparing the distributions of channel orientation among the three sites of interest.

Tuberosity (*Region I*)	θ_{T1}, ϕ_{T1}	θ_{T2}, ϕ_{T2}	θ_{T3}, ϕ_{T3}	θ_{T4}, ϕ_{T4}	θ_{T5}, ϕ_{T5}
p (I-II)	**$2.5\ 10^{-71}$**	**$1\ 10^{-60}$**	**$1.2\ 10^{-55}$**	**$2.7\ 10^{-36}$**	**$2.5\ 10^{-37}$**
Periosteal (*Region II*)	θ_{S1}, ϕ_{S1}	θ_{S2}, ϕ_{S2}	θ_{S3}, ϕ_{S3}	θ_{S4}, ϕ_{S4}	θ_{S5}, ϕ_{S5}
p (II-I)	**$1.4\ 10^{-37}$**	**$3.4\ 10^{-46}$**	**$3.3\ 10^{-47}$**	**$1\ 10^{-71}$**	**$5.8\ 10^{-56}$**

Cortical (*Region III*)	θ_{C1}, ϕ_{C1}	θ_{C2}, ϕ_{C2}	θ_{C3}, ϕ_{C3}	θ_{C4}, ϕ_{C4}	θ_{C5}, ϕ_{C5}
p (III-I)	**1.8 10^{-12}**	**1 10^{-18}**	**2.7 10^{-20}**	**7 10^{-74}**	**2.9 10^{-24}**
Tuberosity (*Region I*)	θ_{T1}, ϕ_{T1}	θ_{T2}, ϕ_{T2}	θ_{T3}, ϕ_{T3}	θ_{T4}, ϕ_{T4}	θ_{T5}, ϕ_{T5}

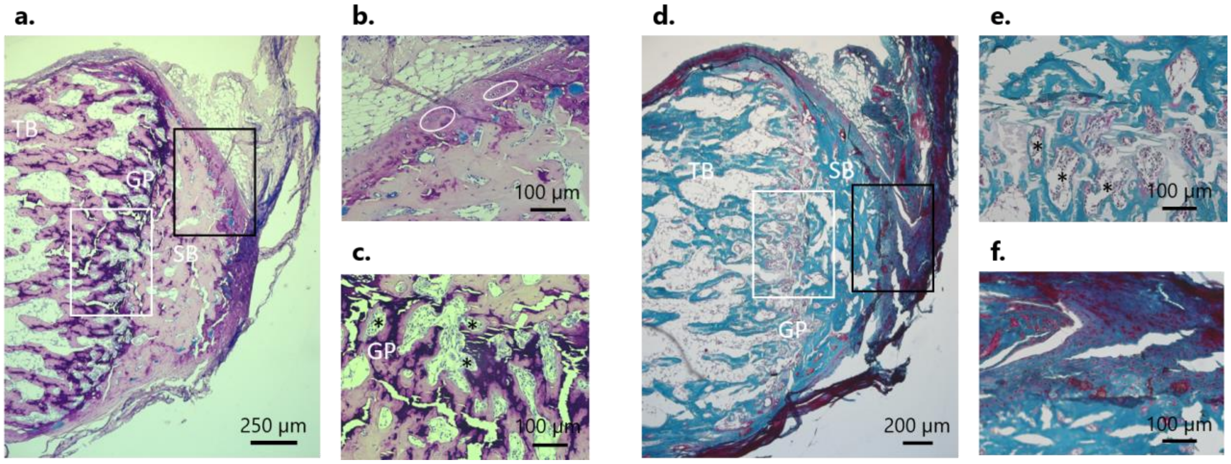

Fig. S4. Histological analysis from two sagittal sections stained with Giemsa and Goldner. TB, GP and SB denote trabecular bone, growth plate and subchondral bone, respectively. The orientation of the cutting plane is slightly different as for the sample reported in Fig. 2. (a) Bright field light microscopy images of a thin section stained with Giemsa. The black and the white frames show the regions depicted with higher magnification in (b) and (c), respectively. (b) Magnified view highlighting a region at the growth plate, with cartilage inclusions (dark violet) and crowded with cells (black asterisks). (c) Zoom in the cartilage region (violet), with some chondrocytes circled in white. (d) Bright field light microscopy images of a thin section stained with Goldner. The black and the white frames show the regions depicted with higher magnification in (e) and (f), respectively. (e) Magnified view of the growth plate highlighting how active the region is: black asterisks show surfaces covered with cells, probably osteoblasts. (f) Zoom in the cartilage region: the darker area is less (or not) mineralized.

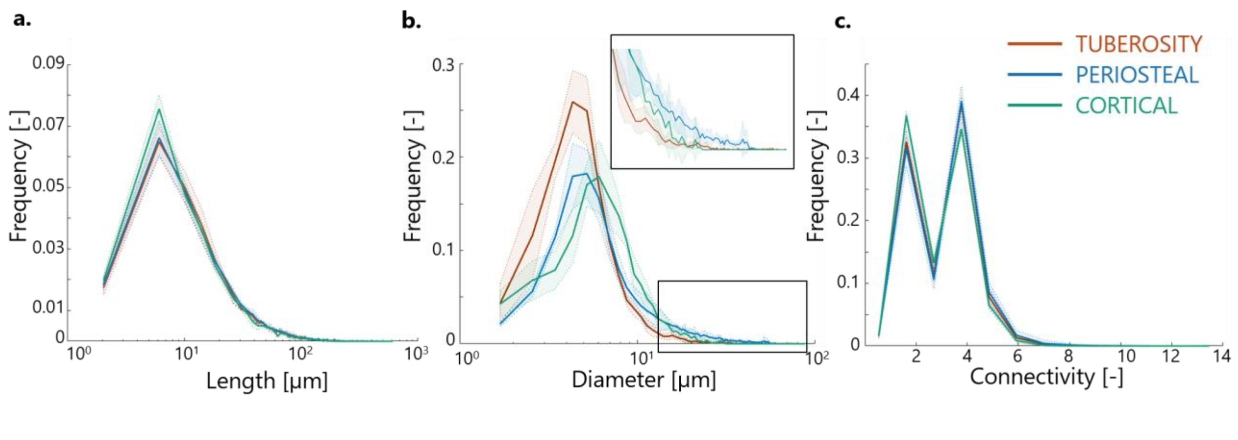

Fig. S5. Three-dimensional local analysis of the channel morphology at the three sites of interest based on the high resolution micro-CT scans. Frequency distributions (normalized to unit area) of the channel length (**a**), diameter (**b**) and connectivity (**c**) for the entire dataset. Data reported as mean value (thick lines) with one standard deviation interval (shaded area).

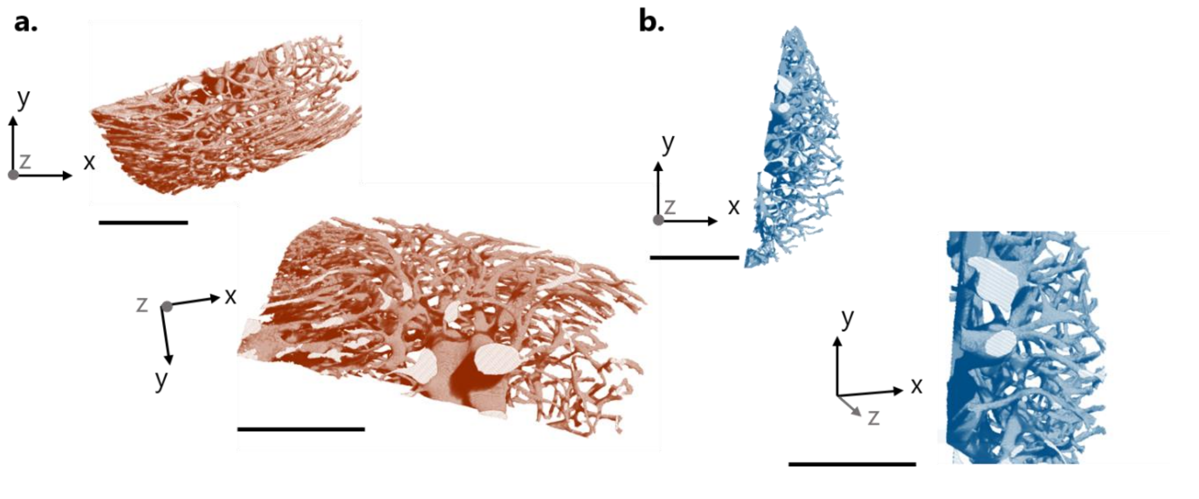

Fig. S6. Illustration of large blood vessels entering the bone and spreading into smaller vessels for two sites of interest based on the high resolution micro-CT scans. In the tuberosity region (**a**), a big central blood vessel unravels into smaller and slender channels arranged like “butterfly” wings. The periosteal bone region (**b**) exhibits intermediate-sized channels lacking a clear predominant orientation. Scale bars: 500 µm.

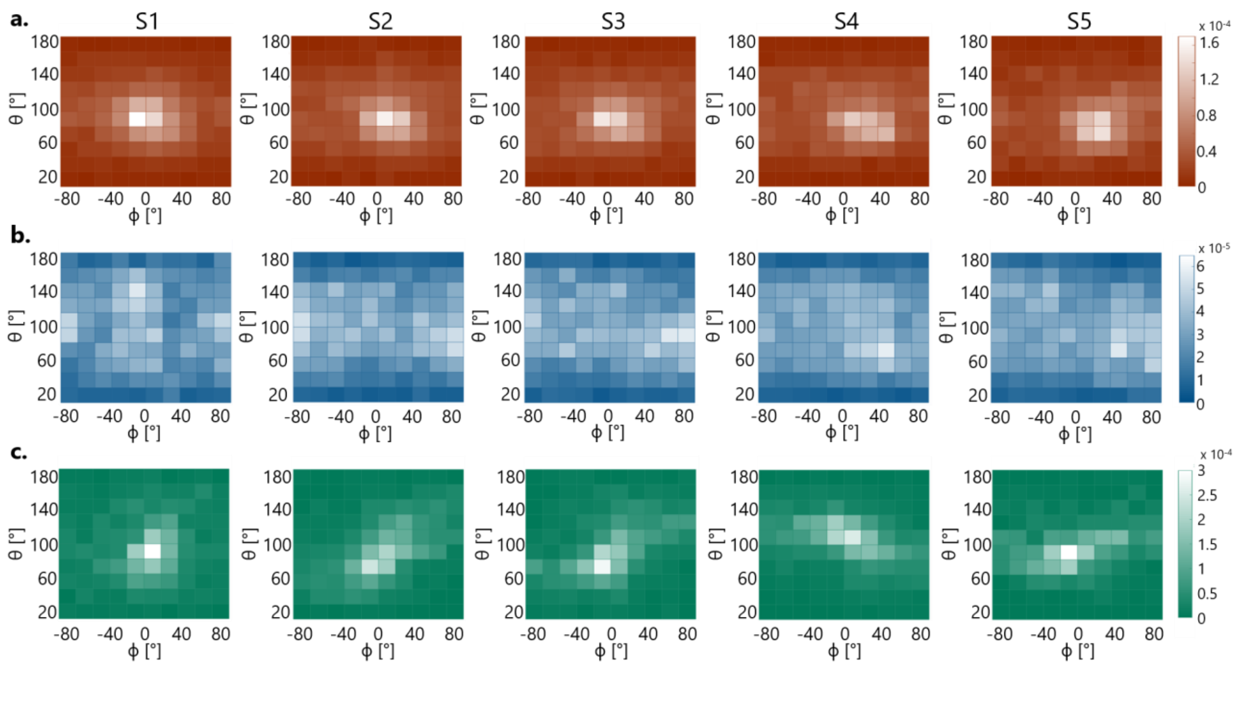

Fig. S7. Three-dimensional local analysis of the channel orientation at the three sites of interest based on the high resolution micro-CT scans. Two-dimensional heat maps of channel orientation for the whole dataset, confirming specific patterns for each region: the tuberosity (**a**) and the cortical bone (**c**) exhibit strong anisotropy whereas the periosteal bone (**b**) is rather isotropic. Data normalized to unit volume.

Table S2

Results of the two-sample two-dimensional Kolmogorov-Smirnov tests comparing the distributions of fibrochondrocyte lacuna orientation between the two sites of interest.

Tuberosity (*Region I*)	θ_{T1}, ϕ_{T1}	θ_{T2}, ϕ_{T2}	θ_{T3}, ϕ_{T3}	θ_{T4}, ϕ_{T4}	θ_{T5}, ϕ_{T5}
p (I-II)	$\mathbf{2.1\ 10^{-74}}$	$\mathbf{9.2\ 10^{-69}}$	$\mathbf{4\ 10^{-77}}$	$\mathbf{5\ 10^{-42}}$	$\mathbf{5\ 10^{-89}}$
Periosteal (*Region II*)	θ_{S1}, ϕ_{S1}	θ_{S2}, ϕ_{S2}	θ_{S3}, ϕ_{S3}	θ_{S4}, ϕ_{S4}	θ_{S5}, ϕ_{S5}

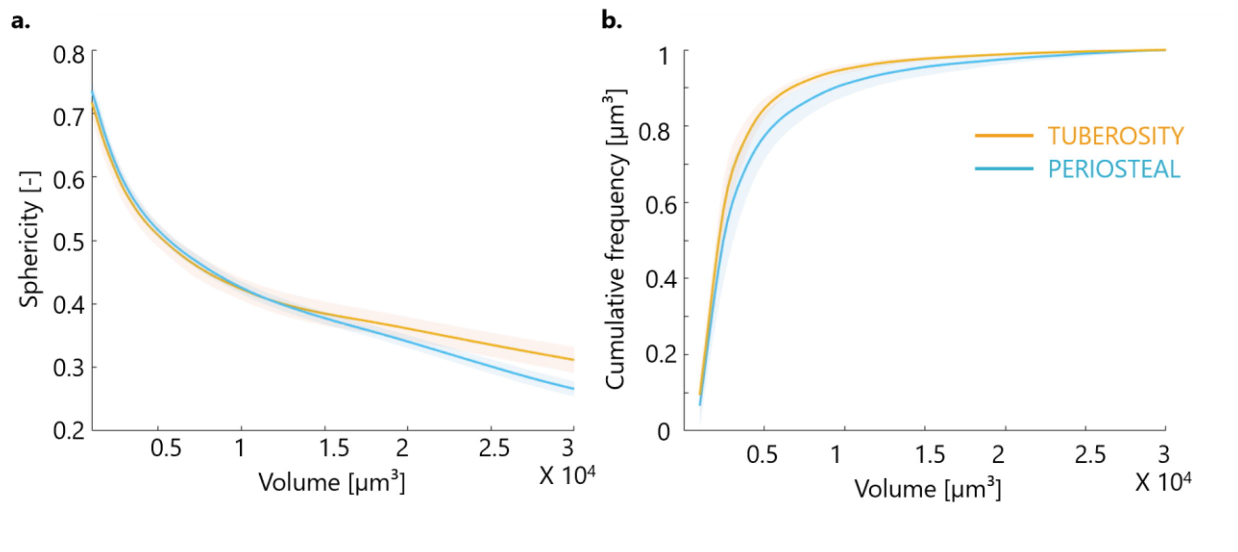

Fig. S8. Three-dimensional local analysis of the lacunar sphericity at the two sites of interest based on the high resolution micro-CT scans. (**a**) Evolution of lacuna sphericity with volume reveals a strong decreasing trend with minor differences between the two sites and only for fairly large volumes. (**b**) Cumulative frequency distribution (normalized to unit area) of lacunae volume showing a sharper transition from small to high volume aggregates within the tuberosity. Data reported for the whole dataset as mean value (thick lines) with one standard deviation interval (shaded area).

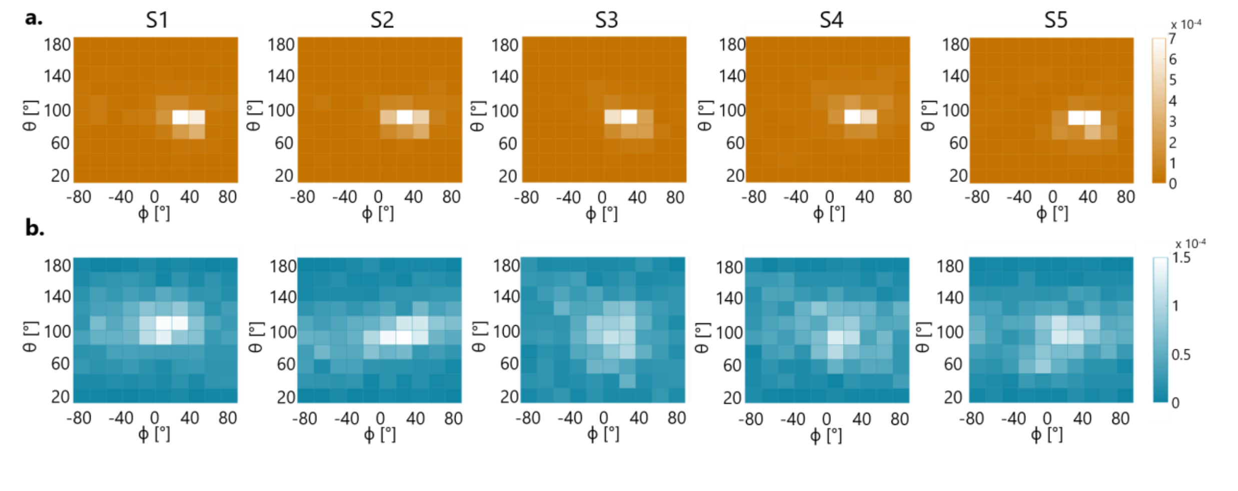

Fig. S9. Three-dimensional local analysis of the lacunar porosity orientation at the two sites of interest based on the high resolution micro-CT scans. Representative two-dimensional heat maps of lacuna orientation for the whole dataset, confirming a clear preferred orientation at the insertion (**a**) compared to the periosteal region (**b**). Data normalized to unit volume.

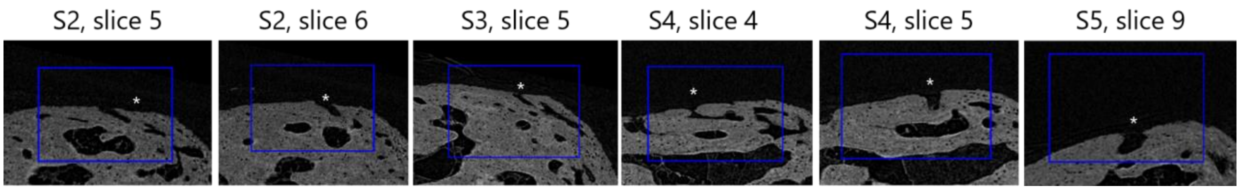

Fig. S10. Roughness of the interface profile line based on the high resolution micro-CT scans. Illustration of the discarded regions for the roughness analysis because of blood vessels entering the bone.

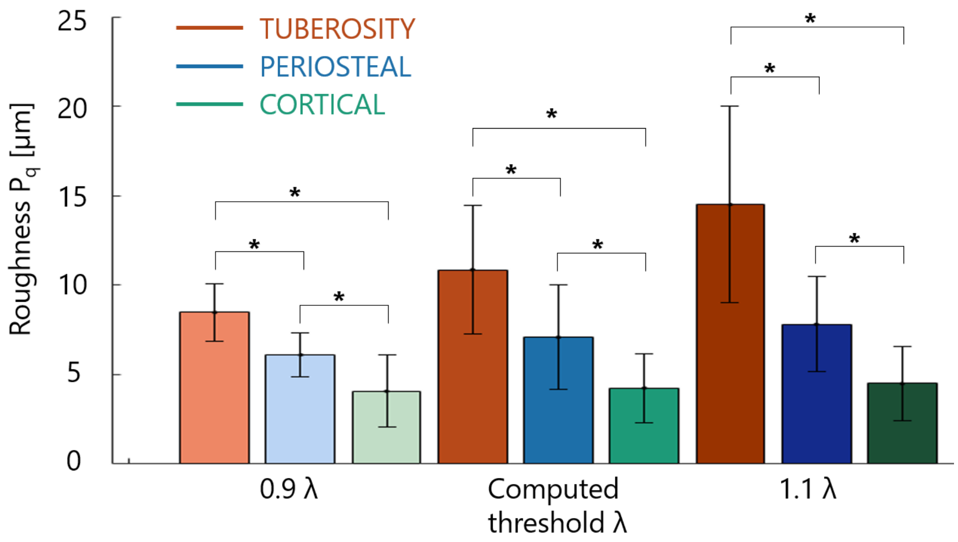

Fig. S11. Sensitivity analysis on the threshold value for roughness computation, conducted for one representative animal (S1). Threshold variation of ± 10% does not influence the statistically significant difference between the three regions of interest.
